# *MetaHarmonizer*: robust biomedical metadata harmonization and a contamination control for inflated LLM performance on public benchmarks

**DOI:** 10.64898/2026.06.13.732088

**Published:** 2026-06-17

**Authors:** Changchang Li, Abhilash Dhal, Kai Gravel-Pucillo, Kaelyn Long, Michele Waters, Ino de Bruijn, Sean Davis, Sehyun Oh

**Affiliations:** 1 Institute for Implementation Science in Population Health, City University of New York School of Public Health, New York, NY, USA; 2 Department of Epidemiology and Biostatistics, City University of New York School of Public Health, New York, NY, USA; 3 Independent Researcher, India; 4 Present address: University of Luxembourg, Esch-sur-Alzette, Luxembourg; 5 Memorial Sloan Kettering Cancer Center, New York, NY, USA; 6 Departments of Biomedical Informatics and Medicine, University of Colorado Anschutz School of Medicine, Denver, Colorado, USA

## Abstract

Public biomedical repositories hold substantial reuse potential, but inconsistent metadata routinely blocks integration across studies. Recent LLM-based harmonization approaches address scale but suffer from non-determinism, hallucinated ontology terms, and, in their highest-accuracy configurations, dependence on proprietary APIs or labeled fine-tuning data. A more fundamental concern is that LLM accuracies on widely-used public benchmarks may substantially inflate transferable capability: under a contamination-controlled evaluation protocol we developed, the apparent LLM-only advantage on the GDC schema-mapping benchmark is inverted and three out of five LLMs recovers 80–100% of GDC identifiers from zero-schema context, suggesting direct memorization. Building on this insight, we present *MetaHarmonizer*, an automated metadata harmonization system designed to be robust by construction: *SchemaMapper* aligns attribute names across schemas, and *OntologyMapper* standardizes values to controlled vocabularies. Both modules implement a multi-stage cascade that escalates to more resource-intensive methods only when earlier stages fall short, with all candidates grounded in pre-defined controlled vocabularies to preclude hallucinated outputs and LLMs used only as bounded preprocessing components rather than inference-time dependencies. On the GDC schema-matching benchmark, *SchemaMapper* with the deployment-optimized LLM-generated alias dictionary achieved 71.6% Top-1 accuracy and the higher Recall@GT than Magneto bipartite variants, recovering significantly more ground-truth mappings; with the best performing alias dictionary, it reached the highest Top-1/Top-5/Recall@GT, and also matched the best Magneto reranker (fine-tuned LLM-reranker) on MRR; and it also outperforms LLM-only performance under contamination-controlled conditions. On four EFO benchmarks, *OntologyMapper* achieved 77.9–95.5% Top-1 accuracy, outperforming *text2term* by up to 16.4 pp and direct LLM inference (against the smaller corpus) by 19.2 pp because memorization is not a viable shortcut for this task. Across both modules, calibrated confidence scores separate correct from incorrect predictions (AUC 0.73–0.94), enabling principled human-in-the-loop triage. Inference is fully local, deterministic, and computationally efficient – seconds on schema mapping and under a minute for ontology mapping of up to ~7,000 terms against the pre-indexed 33,230-term corpus. Released as a Python package with a domain-agnostic architecture, *MetaHarmonizer* provides a scalable foundation for improving the FAIRness of biomedical data and enabling cross-study integration, alongside an evaluation methodology applicable to any LLM-augmented bioinformatics benchmark built on public benchmarks.

## Introduction

The exponential growth of multi-omics data has transformed our understanding of biological systems and disease mechanisms. However, the utility of these vast datasets depends heavily on the quality and accessibility of their associated metadata, which provides crucial context on experimental conditions, patient characteristics, and treatment outcomes. While significant attention has been paid to standardizing and sharing omics data^1–3^, harmonizing patient- and sample-level metadata remains a critical yet often overlooked challenge in the biomedical research ecosystem.

Metadata from public biomedical repositories often exhibits inconsistent terminology, structure, and completeness. This heterogeneity severely impacts the FAIRness (Findability, Accessibility, Interoperability, and Reusability) of valuable research data, making it challenging to perform cross-study analyses or integrate datasets from multiple sources. The situation is particularly difficult for patient/sample-level metadata, where variations in naming conventions, units, and formatting create significant barriers to data identification, integration, and reuse^4,5^.

Computational approaches to metadata harmonization can be organized along two levels: *schema-level alignment*, which maps variable names or column definitions across datasets, and *value-level ontology grounding*, which maps free-text values within those variables to controlled vocabulary terms. The two tasks pose different challenges and have been addressed by largely separate lines of work.

Methods for matching variable names or column definitions have progressed from lexical similarity to neural and embedding-based approaches. Mallya et al.^6^ reported 98.95% Top-5 accuracy using fully convolutional networks with BERT embeddings for cardiovascular variables, although the task was framed as paired-sentence classification across three datasets with rich semantic descriptions rather than open-vocabulary alignment. Salimi et al.^7^ showed that LLM embeddings could support schema harmonization across Parkinson's disease studies, reaching average accuracies above 80%. At the level of phenotype data elements, Pathak et al.^8^ found that 40% still required manual curation, underscoring the residual burden even when computational support is available. Most relevant to the present work, Liu et al.^9^ introduced Magneto, which combines small language models for candidate retrieval with LLM-based reranking and was evaluated on a benchmark of 736 GDC data dictionary attributes; its best-performing variant requires LLM API calls at inference time and depends on domain-specific fine-tuning data.

A larger body of work has tackled the more heterogeneous task of mapping free-text values to ontology terms, where lexical and rule-based methods remain the most widely used strategy. Miotto et al.^10^ processed over 90,000 influenza records using pattern matching, demonstrating early feasibility for structured extraction. Urbanowicz et al.^11^ reported ~85.5% mapping rate through automated exact string matching, and Mate et al.^12^ processed 98.7% of records across 10 European Biobanks. Gonçalves et al.^13^ developed *text2term*, an open-source tool that maps free-text biomedical entity descriptions to ontology terms using Term Frequency-Inverse Document Frequency (TF-IDF)-based cosine similarity and edit distance, achieving 73–81% Top-1 accuracy on curated EFO benchmarks. Hybrid pipelines that incorporate ontology resources extend coverage further; Grossman et al.^14^ reached 94.3–99.6% using MetaMap^15^ and UMLS, but require substantial domain expertise for rule curation and incur an ongoing maintenance burden as terminologies evolve. These studies consistently report failures with typographic variation, ambiguous abbreviations, and non-standardized naming, and plateau at 60–80% automation, leaving a substantial manual harmonization burden.

Embedding- and LLM-based approaches have begun to close this gap recently. Dylag et al.^16^ used Sentence-BERT^17^ clustering to achieve a 117-fold speed improvement over manual curation, but at the cost of moderate accuracy (V-measure 0.237) and limited validation. Higashi et al.^18^ combined LLMs with semantic clustering to harmonize four key attributes across more than 400,000 human gut microbiome samples, and Ikeda et al.^19^ applied LLM-based methods to extract biological terms from over 40 million epigenomics records. The strengths and limits of LLM-based grounding are now well documented: Verbitsky et al.^20^ standardized 691,220 terms across three clinical domains and reported 90–96% accuracy for in-dictionary terms but only 12–17% for out-of-dictionary terms.

Looking across these efforts, several systemic limitations remain unresolved. First, most studies remain confined to a single domain, data type, or repository, and cross-domain evaluation is largely absent. Second, LLM-based approaches, while scalable, still require substantial human oversight and face fundamental challenges: non-deterministic outputs that conflict with FAIR principles^21^, hallucination of plausible but nonexistent ontology terms^22^, limited contextual awareness across thousands of variables^23^, and practical barriers including API costs and data privacy concerns^24^. Third, most approaches address only one facet of the harmonization problem, leaving the full spectrum of curation tasks required for cross-study integration unaddressed, including reconciling conflicting values, consolidating redundant metadata fields, and incorporating study-level context. The accuracy–interpretability tradeoff remains unresolved: high-accuracy neural approaches operate as black boxes, while interpretable lexical methods plateau at insufficient levels of automation. No existing system adequately balances automation completeness, cross-domain robustness, computational efficiency, and interpretability.

To address these limitations, we developed *MetaHarmonizer*, an automated metadata harmonization framework comprising two modules: *SchemaMapper* for column-level alignment and *OntologyMapper* for value-level ontology grounding. *MetaHarmonizer* implements a progressive, multi-stage architecture that balances speed, cost, and accuracy. The framework combines hybrid embedding strategies grounded in real ontology terms with calibrated confidence scores that enable practical human-in-the-loop workflows. Benchmarked against the most directly comparable tools (*Magneto* for schema matching and *text2term* for ontology mapping), *SchemaMapper* achieves competitive or better performance with substantially lower computational cost and no dependence on external LLM APIs at inference time, and *OntologyMapper* displayed superior performance on all metrics used. We further show that *OntologyMapper* outperforms LLM-only inference, and that, under contamination-controlled evaluation, *SchemaMapper* paired with an LLM-generated alias dictionary matches LLM-only performance without incurring inference-time API costs. In the course of this benchmarking, we developed a contamination-control protocol (combining source-side paraphrase, target-side identifier renaming, and direct memorization probes) that quantifies the extent to which LLM-only performance on public schema benchmarks reflects pretraining exposure rather than transferable matching capability. We present this methodology alongside *MetaHarmonizer* as a general-purpose contamination control for LLM-augmented benchmarks.

## Methods

### Architecture

*MetaHarmonizer* comprises two modules that address complementary harmonization tasks: *SchemaMapper* aligns heterogeneous attribute names to a target schema, and *OntologyMapper* standardizes free-text values to controlled vocabulary terms. Both modules implement a multi-stage cascade that escalates from inexpensive exact and lexical matching to embedding-based methods, with all candidates drawn from pre-defined controlled vocabularies; the sections below describe each stage in detail. Within this design, *stages* are sequential steps in the pipeline, and *methods* are alternative or sub-approaches available within a stage. *MetaHarmonizer* is implemented in Python, with FAISS-based vector search, SQLite-based caching, and connectors to external terminology services (NCI, UMLS, OLS).

### Prepare Inputs

There are two required inputs for *MetaHarmonizer*: 1) Queries: column names for *SchemaMapper* and values for *OntologyMapper* that are subject to harmonization, and 2) Targets: a list of target attributes for *SchemaMapper* and a corpus of allowed ontology labels for *OntologyMapper*. Both column names and values are normalized through case normalization, whitespace handling, and removal of special characters. During the input pre-processing for *SchemaMapper*, non-informative values (i.e., *yes/no/unknown*) are also removed from the list of unique values; while they have information on the attribute's status (denoted by the column name), they do not provide any additional information during Stage 2 of the schema mapping pipeline. Removing these values results in the special case (*M* = 0) during Stage 2–2 of *SchemaMapper*. The key parameters for each module are listed in Supplementary Tables 1–2.

#### SchemaMapper

*SchemaMapper* has a cascade architecture with 1) early termination when confidence is high, avoiding unnecessary computation, 2) best-so-far tracking, ensuring a result is always returned even if no threshold is met, and 3) fine-grained control through per-strategy thresholds. We set the default thresholds based on empirical tests using manually created gold-standard datasets^25^ distinct from the benchmark datasets.

##### Stage 1. Exact/Fuzzy.

This stage performs exact and fuzzy matching between the queried column names and the target schema’s attribute names. For fuzzy matching, we used the token_sort_ratio function from the *RapidFuzz* library^26^. There are four method tags for exact (exact) vs. fuzzy (fuzzy) matching against target (std) vs. its alias (alias); std_exact, alias_exact, std_fuzzy, and alias_fuzzy. Aliases can be sourced from manual curation or LLM-based generation (see the section, ‘LLM-generated alias dictionary for SchemaMapper’, below).

##### Stage 2. Column Value Level.

This stage is invoked when the query includes column values. It targets unmatched, non-numeric columns from Stage 1 and uses their unique, non-null data values as inputs. Each value is embedded individually and compared against the embeddings of allowed values from a ‘target attribute + allowed values’ dictionary. For each query, Top-k similar dictionary values vote for their parent field; votes are aggregated per target field using log-compressed frequency weights, and a field is returned when its weighted proportion exceeds the threshold (default: 0.2).

###### 2–1. Allowed Value Matching (method = ‘value’)

This method applies to columns containing a *relatively stable set of categorical values*, such as sex, country, and ethnicity. The algorithm defines a ‘hit’ as any value pair above the threshold (default is cosine similarity >= 0.85). The ‘hit rate’ is calculated for each input column *i* as:

$d_{i}=$ number of unique values in input column i

$n_{i}=$ number of input values that achieve 'hiť status (cosine similarity >= 0.85 with any target value)

$p_{i}=$ hit rate

(2.1) $$p_{i}=\frac{n_{i}}{d_{i}}$$

In the special case where $d_{i}=0$ (e.g., values are limited to *yes/no/unknown* and are removed during preprocessing), we set $p_{i}=0$ and note that there is insufficient evidence. Input columns are ranked by $p_{i}$ in descending order. When multiple columns have identical $p_{i}$, they are ordered by their average similarity scores.

###### 2–2. NCIt Voting Method (method = ‘ontology’)

This method is triggered for columns where possible values are actively changing or growing, such as treatment names and diseases. We map unique values from each column to ontology terms using the NCIt client, leveraging NCIt’s built-in fuzzy matching and synonym/alias expansion. Then we traverse the NCIt hierarchy upward to determine whether mapped terms belong to predefined ontology nodes per categories. The examples of predefined categories include ‘Body Part’ (NCIT:C32221) for body_site, ‘Disease or Disorder’ (NCIT:C2991) for disease, and ‘Pharmacologic Substance’ (NCIT:C1909) for treatment_name.

M= total number of input values successfully assigned to NCIt terms

$m_{i}=$ number of values assigned NCIt terms that match the target category $c_{i}$

$v_{i}=$ NCIt voting score/proportion for category $c_{i}$

(2.2) $$v_{i}=\frac{m_{i}}{M}$$

If M=0 (no values successfully mapped to NCIt terms), then $v_{i}=0$ and the result is noted as 'no NCIt evidence'. The score $v_{i}$ represents the proportion of successfully mapped values that belong to the target category $c_{i}$, providing a confidence measure for categorizing the input column.

##### Stage 3. Column Name Level.

This stage contains two methods for different value types (numeric vs. categorical).

###### 3–1. Numeric Column Matching (method = 'numeric')

This method is used if a column predominantly contains numeric values, verified by numeric-type checks and unit inference. For example, columns containing numbers followed by "*years/months/days*" are classified as "time" type, while those followed by "*mg/ml/AUC*" are classified as "dose" type. The similarity score for numeric fields combines two components: the primary component is the cosine similarity between sentence transformer embeddings of the query field (e(q)) and candidate field (e(ci)), and the second component is family boost, an additional factor applied when both query and candidate columns belong to the same type. $\beta_{\text{family}}$ is a weighting parameter that controls the strength of the family boost, and I is an indicator function that returns 1 when the query and candidate belong to the same family, and 0 otherwise.


(2.3) $$s_{i}=cos\left(e(q),e\left(c_{i}\right)\right)+\beta_{\text{family}}I\left(q,c_{i}\right)$$

###### 3–2. Semantic Column Matching (method = ‘semantic’)

This stage processes unseen or ambiguous non-numeric column names that failed to match in Stage 2, as well as low-confidence matches from Stage 3–1. The alias dictionary (the same one used in Stage 1) for target columns can be used in this stage as well. It computes semantic similarity between the query and the target/alias column names.

### LLM-generated alias dictionary for *SchemaMapper*

To improve schema mapping performance, we expand each target field into plausible surface forms provided through an alias dictionary. *SchemaMapper* uses this dictionary both as a string index (for rule-based matching at Stage 1) and as an embedding index (for semantic retrieval at Stage 3). Aliases for each target attribute were generated from five structured passes, each targeting a distinct class of surface variation hinted from the previous observation^25^:

1. Synonym pass: alternative natural-language phrasings (e.g., vital_status → survival status).
2. Abbreviation pass: acronyms and abbreviated forms commonly used in clinical and research databases (e.g., lymph_nodes_examined_positive → LN+, LNs_pos).
3. Value-encoded boolean indicators (e.g., ADJUVANT_CHEMO → treatment_type).
4. Composite pass: fields with embedded modifiers or qualifiers (e.g., PRIOR_, BASELINE_, AGE_AT_).
5. Institutional pass: plausible institution- or consortium-specific variants (e.g., TCGA_SUBTYPE, CPTAC_TUMOR_GRADE).

Each pass operated on batches of 20 fields and returned a structured CSV with columns source, field_name, and is_numeric_field. We tested four Anthropic models via API (claude-opus-4–7, claude-sonnet-4–5, claude-haiku-4–5, claude-opus-4–5), two Google API models (gemini-2.5-flash, gemini-2.5-pro), and three open-weight models via local Ollama inference (gemma3:27b, gemma4:26b, and qwen3:32b) (Supplementary Table 9). Outputs from all five passes were concatenated, deduplicated on the ‘source:field_name’ pair, and screened for hallucinated field names (i.e., aliases generated for fields not present in the input schema) that were removed before downstream use. The alias dictionary is target-schema-specific and requires no labeled training data; only the target field names are needed to generate it.

The alias dictionary augments two schema-matching stages: In Stage 1, *SchemaMapper* performs a normalized lookup over the alias index and applies *RapidFuzz* token-sort-ratio matching against the alias set when no exact hit is found. This is cascaded immediately after its standard dictionary counterparts. In Stage 3, *SchemaMapper* fuses Top-*k* retrievals from the standard field-name index with Top-*k* retrievals from the alias index, using pre-computed sentence-transformer embeddings of all alias strings. Stage 2 (value-based) does not consume the alias dictionary.

#### OntologyMapper

Our ontology mapping module employs a progressive multi-stage pipeline, where each stage handles increasingly complex matching scenarios. Once a term is resolved at a given stage, it is removed from the candidate pool and not re-evaluated by downstream stages.

##### Index Construction.

We prebuild a local ontology term corpus from selected root ontology terms. The corpus contains all descendants of the root, including label, OBO ID, description, and synonyms. From this corpus, we construct per-category SQLite tables used by Stage 2.5; terms are fetched once per category from the NCI EVS REST API (for NCIt) or EBI OLS4 API (for non-NCIt). Alternatively, the tables can be populated offline from a pre-saved corpus JSON. A paired FAISS index is then built over the synonym rows, so each FAISS vector ID equals its SQLite row ID.

##### Stage 1. Exact Matching.

This stage attempts a direct lookup of each query against the ontology corpus without expansion.

##### Stage 2. Semantic Matching.

Queries unresolved by Stage 1 are passed to Stage 2, which performs vector retrieval over the same ontology corpus. Two preprocessing steps are applied to the residual queries before embedding, both implemented as query-side transformations - lightweight normalization and shortname-to-fullname expansion. The transformed queries and the corpus are embedded with a biomedical encoder. We use SapBERT^27^ as the default model in this stage. SapBERT is primarily trained on the Unified Medical Language System (UMLS), a massive metathesaurus and collection of biomedical ontologies containing over 4 million concepts and 10 million+ synonym pairs. We implement two alternative methods (the default is LM) for generating text representations. *OntologyMapper* provides two interchangeable Stage 2 backends: a sentence-transformer pipeline (ST) that uses mean-pooled token embeddings, and a language-model pipeline (LM) that uses CLS-token embeddings (no statistically significant difference was observed in the tests conducted in this study). In both cases, query and corpus vectors are L2-normalized, and the Top-k corpus entries are retrieved per query by cosine similarity. Each candidate is returned with its similarity score in [0, 1] and assigned a match level of 2.

##### Stage 2.5. Synonym Dictionary Boost.

Stage 2.5 targets results from Stage 2 whose Top-1 scores fall below the threshold (default: 0.9). For each such query, it retrieves Top-k matches from an FAISS-indexed ontology synonym dictionary, merges them with the Stage 2 candidates (taking max score per duplicate), and re-ranks. The Stage 2 row is replaced with the merged ranking only if the new Top-1 score is higher or the curated term reaches a better match level; otherwise, Stage 2 is left untouched.

##### *SchemaMapper* Benchmarking

We benchmarked *SchemaMapper* against Magneto (version: 0.3.0.dev0)^9,28^, an end-to-end schema-matching system. We used four reported Magneto variants: zero-shot (zs) and fine-tuned (ft) versions with bi-encoder (bp) and fine-tuned LLM (llm) re-rankers (Magneto-zs-bp, Magneto-ft-bp, Magneto-zs-llm, and Magneto-ft-llm). We reproduced the Magneto bipartite (BP) graph reranker in both zero-shot (zs) and fine-tuned (ft) configurations using the published code and benchmark data. For the Magneto LLM reranker variants, we report the values from the original paper, as these require access to the proprietary LLM API. The target benchmark data were 10 Clinical Proteomic Tumor Analysis Consortium (CPTAC) studies previously used by Magneto. Each study contains heterogeneous metadata column names, which were manually curated by domain experts into 736 standardized target attributes in the GDC data dictionary. Across the 10 studies, 165 source–target pairs had curated ground-truth annotations^28^. Ground-truth annotations include one-to-many mappings, where a single source column maps to multiple acceptable targets.

##### *OntologyMapper* Benchmarking

We compared *OntologyMapper*’s performance against *text2term* (v4.1.2)^13^, a widely used tool for mapping free-text descriptions of biomedical entities to ontology terms. We used benchmark datasets previously established for *text2term*: (1) The UKBB-EFO *benchmark* (n = 888) consists of mappings between UK Biobank phenotype descriptions and EFO terms, derived from the curated mapping set described by Sollis et al.^29^. This dataset represents the most challenging scenario, as UK Biobank phenotype descriptions are often colloquial, abbreviated, or otherwise divergent from formal ontology labels. (2) The Biomappings-EFO *benchmark* (n = 795) draws from the Biomappings community-curated collection of cross-ontology mappings^30^, filtered to those targeting EFO. These mappings use source ontology term labels as input strings, providing a scenario in which source terms are structured ontology labels rather than free text. (3) The OLS-EFO benchmarks use cross-ontology mappings hosted in the EMBL-EBI Ontology Lookup Service (OLS). We evaluated two subsets: *a disease-restricted subset* (n = 5,770 for *OntologyMapper*; n = 5,824 for *text2term*) and the *full cross-ontology mapping corpus* (n = 7,377 for *OntologyMapper*; n = 7,504 for *text2term*). As with Biomappings-EFO, source terms are ontology labels. Query-count differences between tools reflect divergent handling of rows that share a query string but map to different targets; see ‘Denominator conventions across analyses’ section in the Supplementary Methods for the per-analysis denominator scheme.

For the head-to-head benchmark evaluation, we constructed a merged reference corpus for *OntologyMapper* rooted in the Experimental Factor Ontology (EFO, v3.62.0). We extracted 51,862 native Compact Uniform Resource Identifiers (CURIEs) from the EFO OWL distribution. Restricting to the twelve ontologies that EFO cross-imports (EFO, MONDO, Orphanet, CHEBI, HP, UBERON, CL, GO, OBI, PATO, DOID, and BFO) and flattening imported terms to the EFO namespace yielded 39,460 rows compatible with *OntologyMapper*’s single-source partitioner. The module's default obsolete filter then removes rows whose label begins with obsolete_, leaving 33,230 live terms used for retrieval. Corpus composition is EFO-dominated (16,218 terms; 48.8%) with substantial contributions from MONDO (9,508; 28.6%), Orphanet (2,051; 6.2%), CHEBI (1,722; 5.2%), and HP (1,591; 4.8%); UBERON, CL, GO, OBI, PATO, DOID, and BFO together account for the remaining 6.4%. Synonyms were extracted in parallel, producing a synonym index of 142,645 rows covering exact, related, narrow, and broad synonym axiom types. We used the same 12-ontology EFO corpus (v.3.62.0) as a target corpus for *text2term*. Due to different parsing strategies, this same target corpus resulted in 33,659 terms (~1.3% margin); the additional 429 terms are mostly label-less or duplicate entries irrelevant to actual mapping.

### Runtime Analysis

For *SchemaMapper* runtime analysis, each benchmark was timed under two conditions: 1) End-to-end per study wraps a fresh mapping module instantiation – SentenceTransformer load, target-schema encoding, and Stage 1/2/3 retrieval over every source column. 2) Per-column, per-stage records wall-clock time for each (column, stage) call. *SchemaMapper* does not persist an embedding index between runs: target-schema and alias embeddings are recomputed within each module instantiation, so the initialization cost is paid once per study rather than amortized across a disk cache. All timings were collected on a single workstation: Apple M4 Pro, 48 GB unified memory, internal SSD, macOS Sequoia 15.6.1 (aarch64); Python 3.13.5, PyTorch 2.8.0 (CPU-only; the MPS backend was not used), sentence-transformers 5.1.1. Embedding inference was executed on a CPU with single-threaded BLAS (Basic Linear Algebra Subprograms).

Each *OntologyMapper* benchmark was timed under three conditions: 1) *Cold* measures a first-time *OntologyMapper* run at a previously unseen corpus (available as a local OWL file); it includes corpus FAISS embedding, synonym FAISS embedding, and Stage 1/2/2.5 retrieval. 2) *Warm* measures a repeat *OntologyMapper* run that reuses cached FAISS indices and executes Stage 1/2/2.5 retrieval. 3) *text2term* measures a single invocation per benchmark, with ontology parse amortized across benchmarks within a session. Warm runs reuse a three-layer cache: SQLite concept tables, an on-disk FAISS index, and in-process model weights that are populated once per (model, corpus-content) pair. Timings used the same workstation as *SchemaMapper*, with FAISS 1.11.0 and *text2term* v4.1.2 added.

### Performance Evaluation

We adopted four evaluation components.

1. ***Top-k accuracy*** (k = 1,3,5) measures the proportion of queries for which the correct target column appears among the Top-k returned candidates. For queries with multiple acceptable ground-truth targets, we applied the “any-match” criterion, counting a prediction as Top-1 correct if the Top-1 prediction matched any acceptable target.
2. ***Mean Reciprocal Rank (MRR)*** reports the average inverse rank of the first correct match, rewarding systems that place correct matches near the top.
3. ***Recall at Ground Truth size (Recall@GT)***, a standard metric from the Valentine schema matching benchmar^9,31k^ and adopted by Magneto^9,31^, measures the proportion of acceptable ground-truth targets recovered within the Top-G candidates, where G is the number of the ground-truth matches per query; when G = 1, Recall@GT reduces to Top-1 accuracy, and when G > 1, it captures the system's ability to retrieve all valid correspondences. Unlike Top-k and MRR, which depend only on within-query rankings, Recall@GT admits two non-interchangeable formulations – per-query and global – that differ in whether scores must be calibrated across queries; full definitions and the conditions under which each is computable are given in Supplementary Methods.
4. ***Confidence calibration*** assesses whether confidence scores discriminate correct from incorrect Top-1 predictions, since downstream human-in-the-loop review depends on calibrated uncertainty. Confidence distributions for correct versus incorrect Top-1 predictions were compared using Wilcoxon rank-sum tests, with Cohen’s d for effect size and area under the receiver operating characteristic curve (AUC) as a single-number summary of discriminative capacity (correctness as binary outcome, confidence as predictor). Holm correction was applied across strata.

When evaluating multiple studies, Top-k, MRR, and Recall@GT can be aggregated across studies either by pooling all individual queries (*micro*-average) or by computing the metric within each study and averaging them (*macro*-average); macro-averaging preserves the study-to-study variability, while micro-averaging would mask it. We reported micro-averages by default and used macro-averages only for benchmarking against Magneto, where the original paper reported per-study mean across the studies^9^ (Supplementary Methods).

## Results

### Performance of *SchemaMapper* with Alias Dictionaries

During *SchemaMapper* algorithm design, we observed that the target attributes expanded with their manually-curated alias dictionary performed better than the no_alias. To ensure the scalability of *SchemaMapper*, we systematically generated target alias dictionaries using different LLM models. Six proprietary alias dictionaries improved Top-1 *SchemaMapper* accuracy over the no_alias baseline by 12.1–21.8 pp (Supplementary Results and Supplementary Table 3), but the performance among them did not scale with model capability or dictionary size. Although *SchemaMapper* with Opus-4.5-alias produced the highest point estimates (Top-1 = 75.76%, Top-5 = 86.06%, MRR = 0.800, Recall@GT = 0.699, micro-average), it was statistically indistinguishable from five other top-tier dictionaries on Top-1 and Recall@GT (Supplementary Results). Within this equivalence set, Claude Haiku 4.5 sat on the Pareto frontier of cost-versus-accuracy, especially with one-time API cost roughly 20× lower than Opus 4.5 and ~2.8x faster wall-clock time; two Tier-A models (Sonnet 4.5 and Opus 4.7) were each strictly Pareto-dominated by a cheaper same-vendor alternative (Supplementary Figure 1D and Supplementary Table 15). The open-weight Gemma 3 27B dictionary performed at the no-alias baseline; two additional open-weight models (Gemma 4 26B and Qwen3 32B) failed format compliance and produced no usable dictionary.

**Figure 2.**
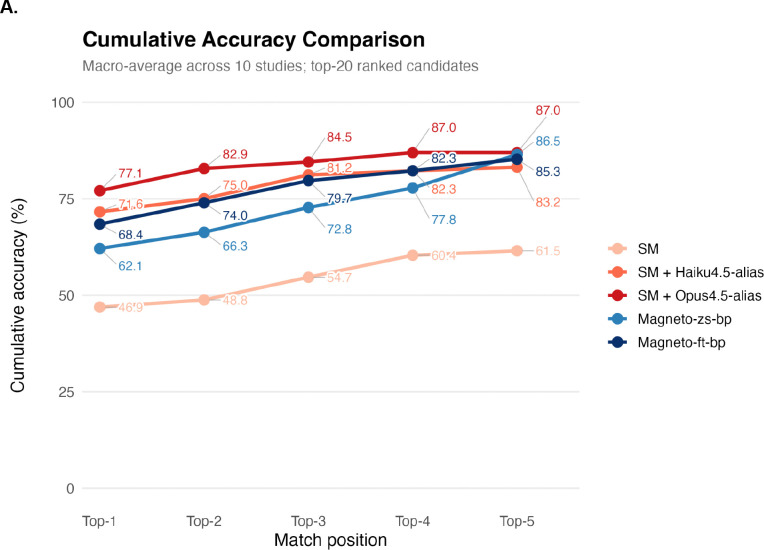

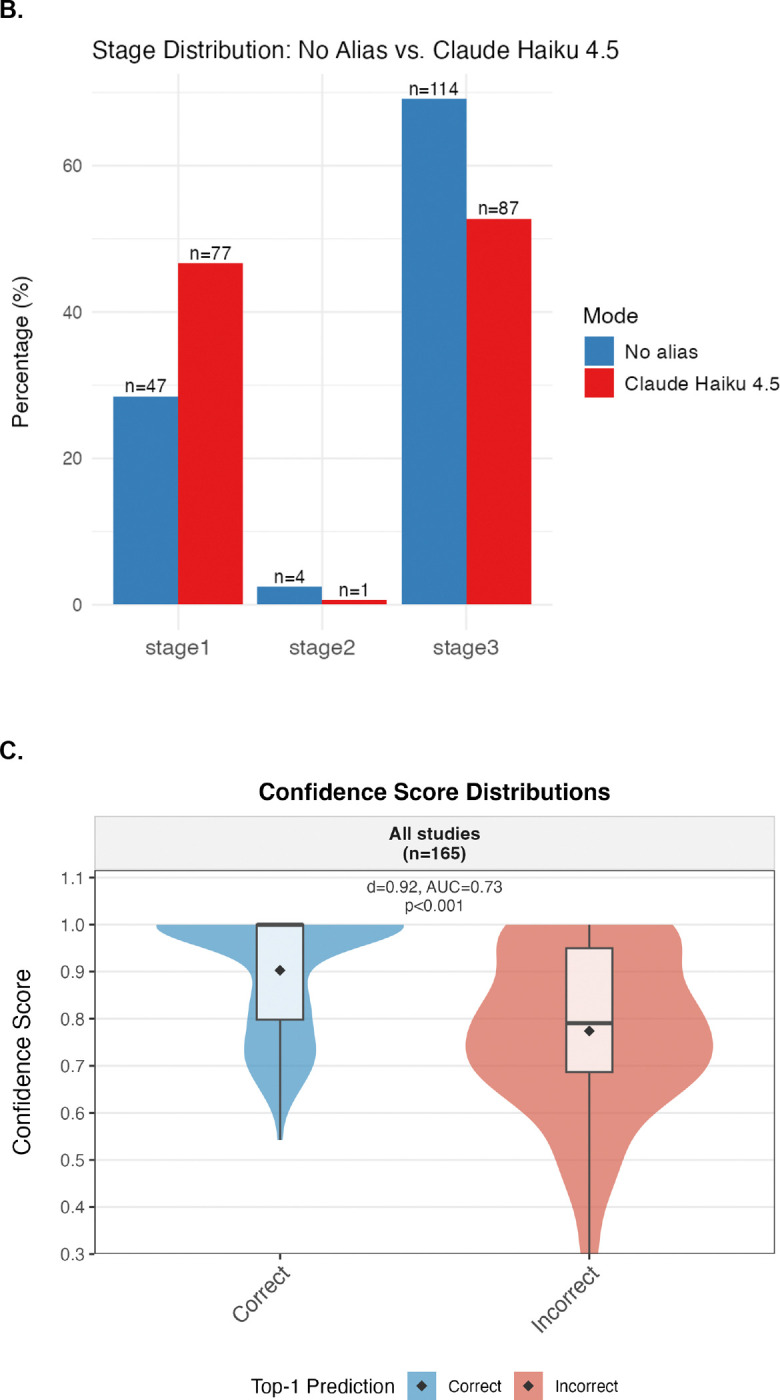
Performance of *SchemaMapper* on the GDC schema mapping benchmark We benchmarked *SchemaMapper* against 165 ground-truth column mappings from the GDC schema matching data. **(A)** Top-*k* accuracy (k = 1–5) for five configurations: Magneto zero-shot bipartite reranker (Magneto-zs-bp, blue), Magneto fine-tuned bipartite reranker (Magneto-ft-bp, navy), *SchemaMapper* without an alias dictionary (SM, peach), and *SchemaMapper* with a Haiku-4.5-alias (SM + Haiku4.5-alias, orange) and an Opus-4.5-alias (SM + Opus4.5-alias, red) **(B)** Stage distribution of all queries with and without the Haiku-4.5 alias dictionary. The dictionary expands Stage 1 coverage from 47 to 77 queries and reduces Stage 3 coverage from 114 to 87 queries; Stage 2 query counts are barely changed. The shift indicates that the alias dictionary resolves additional queries through low-cost lexical matching that would otherwise require semantic embeddings. **(C)** Confidence calibration for the SM + Haiku4.5-alias. Paired violin and box plots compare the score distribution of correct (blue, n = 121) and incorrect (red, n = 44) Top-1 predictions. Correct predictions concentrate near 1.0 (mean = 0.90, median = 1.00), whereas incorrect predictions are more dispersed (mean = 0.77, median = 0.79), yielding strong separation (Cohen's *d* = 0.92, AUC = 0.73, Wilcoxon-p < 0.001). The score is therefore well calibrated for triaging predictions to human review. Panels B and C used SM with Haiku4.5-alias because it is the recommended model for deployment. SM = *SchemaMapper*.

*SchemaMapper* performance also varied by pipeline stage (Figure 2B and Supplementary Table 4): Stage 1 resolved 77 queries at 87.0% Top-1 accuracy, while Stage 3 handled 87 queries at 62.1% Top-1 accuracy. Without aliases, queries previously resolved at Stage 1 fell through to Stage 3 semantic matching, increasing the Stage 3 query pool from 87 to 114 and diluting its within-stage accuracy (Top-1: 62.1% → 38.6%; MRR: 0.692 → 0.495). This demonstrates that the alias dictionary improves performance through two mechanisms: directly, by resolving queries at the most reliable pipeline stage, and indirectly, by reducing the difficulty of the residual semantic-matching pool.

### *SchemaMapper* Benchmarking

We benchmarked *SchemaMapper* with Haiku-4.5-alias, the recommended deployment option, against four Magneto configurations on the GDC schema-matching benchmark (Table 1 and Figure 2A). *SchemaMapper* outperformed both reproducible Magneto bipartite variants (zs-bp and ft-bp) on Top-1, MRR, and per-query Recall@GT: Top-1 accuracy (71.6% vs. 62.1% and 68.4%), MRR (0.763 vs. 0.731 and 0.757), and per-query Recall@GT (0.65 vs. 0.537 and 0.60), with the difference against zs-bp reaching statistical significance (p = 0.033, 0.042, and 0.013). With the best-performing alias dictionary (Opus-4.5-alias), *SchemaMapper* was significantly better than both Magneto bipartite variants on Top-1, MRR, and per-query Recall@GT (p = 0.032, 0.03, and 0.014 for zs-bp; p = 0.03, 0.033, and 0.032 for ft-bp); it is also statistically indistinguishable from Magneto LLM-reranked variants on MRR (per-study paired Wilcoxon-p = 0.93 (zs-llm) and 0.43 (ft-llm)). Per-query Recall@GT complements MRR for this benchmark, where 40% of queries (66/165) have multi-target ground truth. *SchemaMapper*'s confidence scores were well calibrated for distinguishing correct from incorrect predictions (Figure 2C): mean confidence was 0.903 for correct predictions (n = 121) and 0.774 for incorrect predictions (n = 44), with Cohen's d = 0.92 and AUC = 0.73.

### *OntologyMapper* Benchmarking

**Figure 3.**
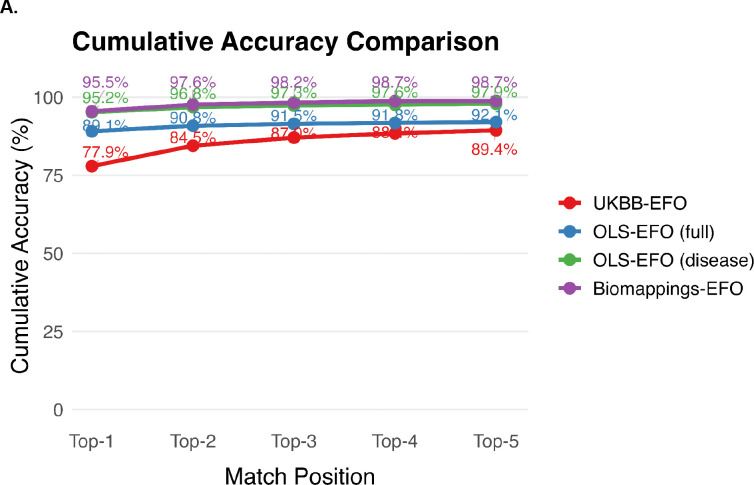

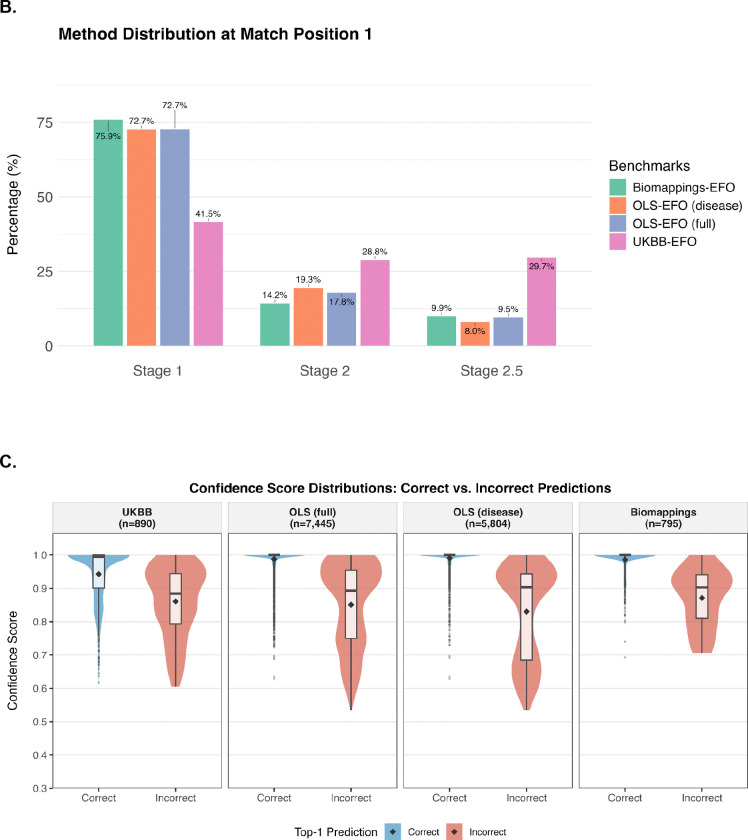
Performance of *OntologyMapper* across diverse benchmarking datasets We evaluated *OntologyMapper* on four benchmarks: Biomappings-EFO, OLS-EFO (disease subset), OLS-EFO (full), and UKBB-EFO. **(A)** Top-k cumulative accuracy (k = 1–5) on each benchmark. The gap between Top-1 and Top-5 accuracy is the largest for UKBB-EFO. **(B)** Composition of correct Top-1 predictions by resolving stage, stratified by benchmark. Stage 1 resolved the majority of queries on benchmarks with formal ontology labels as input (Biomappings-EFO, OLS-EFO disease, and OLS-EFO full), whereas UKBB-EFO depends more heavily on the embedding-based stages (Stages 2 and 2.5), consistent with the greater lexical divergence of UK Biobank phenotype descriptions from EFO labels. **(C)** Confidence score distributions for correct (blue) and incorrect (red) Top-1 predictions, faceted by benchmark (*n* shown above each panel). Paired violin and box plots show the full density of scores; embedded box plots indicate the median (horizontal line), interquartile range (box), and 1.5× IQR whiskers; diamond markers indicate the mean. The prominent density spike at 1.0 among correct predictions reflects Stage 1 terms resolved with perfect confidence. The consistent separation across all benchmarks indicates that confidence thresholding can effectively triage predictions for human review.

*OntologyMapper* consistently outperformed *text2term* across all four benchmarks on both Top-1 and MRR (Figure 3A and Table 2). The largest margins were on Biomappings-EFO (Top-1 95.5% vs. 79.1% (Δ = +16.4pp); MRR 0.969 vs. 0.848), followed by OLS-EFO full (Top-1 89.1% vs. 79.2% (Δ = +9.9pp); MRR 0.903 vs. 0.813). On UKBB-EFO, *OntologyMapper* reached 77.9% Top-1 vs. *text2term*'s 71.6% (MRR 0.826 vs. 0.765). The narrowest gap was on OLS-EFO disease (Top-1 95.2% vs. 93% (Δ = +2.2pp); MRR 0.963 vs. 0.952), the easiest of the four benchmarks, where both tools approach the ceiling set by the curated ground truth.

The multi-stage pipeline contributed differentially across benchmarks (Supplementary Table 5). Stage 1 resolved 32.4–72.5% of queries (median ~67%), accounting for 41.5–75.9% of correct Top-1 predictions (Figure 3B). Among queries routed to Stage 2, Top-1 accuracy ranged 70.4–88.6% at Top-1, and Stage 2.5 resolved queries unmatched by either lexical or semantic match at 59.2–81.5% Top-1.

Confidence scores showed statistically significant separation between correct and incorrect Top-1 predictions across all four benchmarks (Wilcoxon rank-sum test, all p < 2 × 10^−16^ after Holm correction; Figure 3C and Supplementary Table 6). Effect sizes were large, with Cohen's d ranging from 0.954 (UKBB-EFO) to 3.89 (OLS-EFO disease). The AUC for confidence-based discrimination of correct versus incorrect predictions ranged from 0.766 (UKBB-EFO) to 0.935 (OLS-EFO disease), indicating that confidence scores provide a useful signal for identifying predictions that may require manual review. Mean confidence was substantially higher for correct than for incorrect predictions across all benchmarks: 0.94 vs. 0.86 (UKBB-EFO), 0.99 vs. 0.85 (OLS-EFO full), 0.99 vs. 0.83 (OLS-EFO disease), and 0.99 vs. 0.87 (Biomappings-EFO). The lower AUC on UKBB-EFO is consistent with the greater lexical diversity of UK Biobank phenotype descriptions, which span a wider range of difficulty levels, compressing the confidence distribution.

### Runtime Analysis

To evaluate the computational efficiency, we profiled *SchemaMapper* and *OntologyMapper* across their respective benchmark tasks, measuring end-to-end pipeline time and scalability with respect to input size.

**Figure 4.**
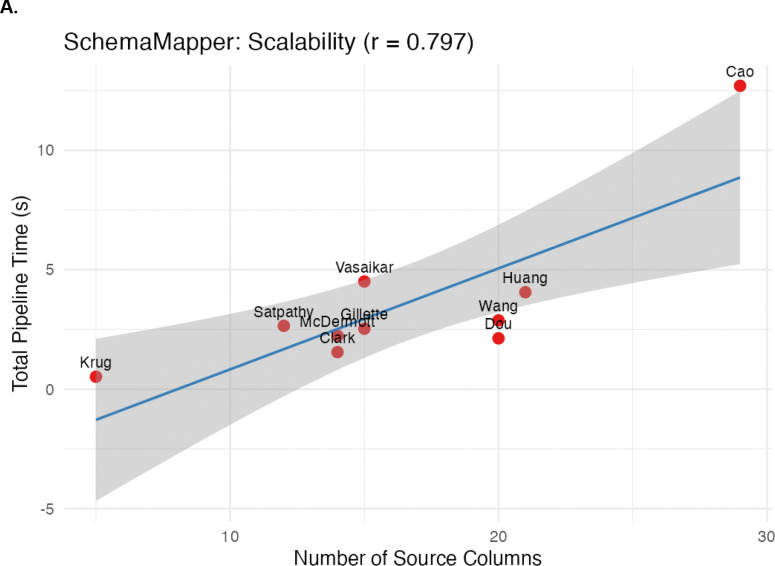

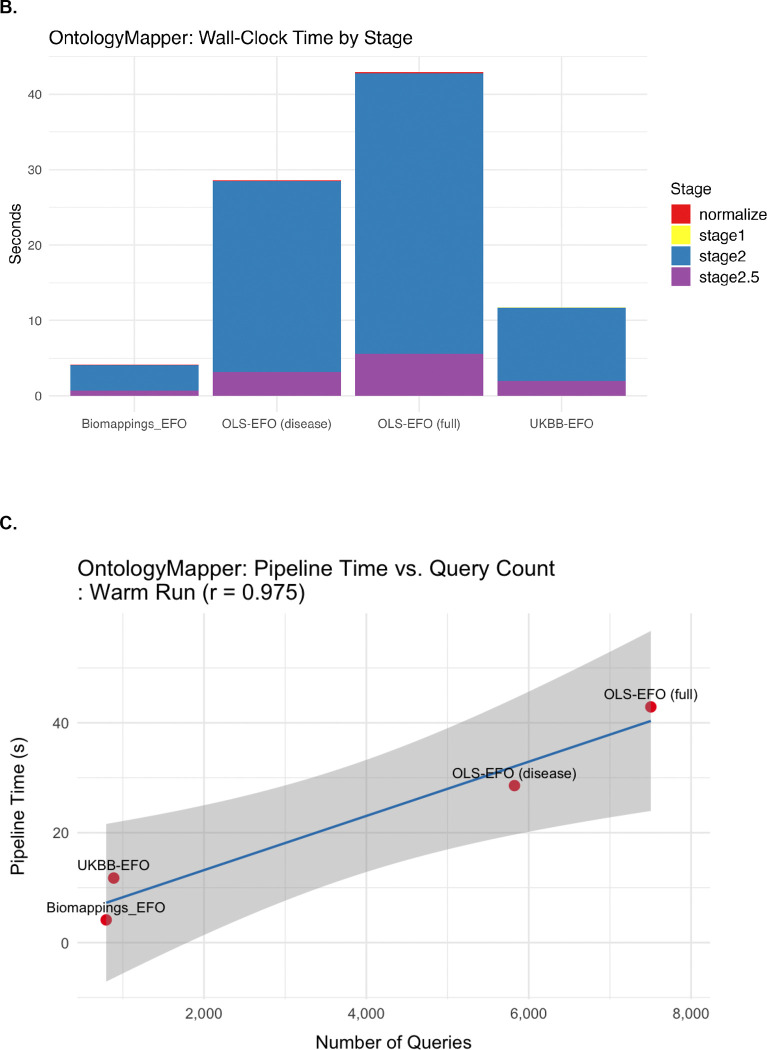
Runtime analysis of *MetaHarmonizer* We profiled the runtime of *MetaHarmonizer* modules on representative benchmarks. The target corpus contains 736 GDC columns for *SchemaMapper* and 33,230 EFO terms for *OntologyMapper*. **(A)** *SchemaMapper* runtime (with the Haiku-4.5-generated alias dictionary) scales approximately linearly with the number of source columns. Each point represents one of the 10 CPTAC benchmark studies (labeled by first author) and plots total pipeline time (seconds) against the number of source columns. The blue line is a linear regression fit with 95% confidence interval (grey shading) (Pearson *r* = 0.797). **(B)** *OntologyMapper* wall-clock time broken down by stage, on each of the four EFO benchmarks. *OntologyMapper* Stages 2 and 2.5 together account for nearly all active pipeline time; Stage 1 contributes negligibly. **(C)** *OntologyMapper* warm-run scaling across the four EFO benchmarks. "Warm" denotes a run in which the FAISS index is already loaded into memory and embeddings are cached, so per-query cost reflects steady-state performance rather than initialization. Pipeline time scales approximately linearly with query count (Pearson *r* = 0.975).

The GDC benchmark datasets for *SchemaMapper*+Haiku-4.5-alias include 10 studies ranging 5–29 source columns each. The multi-stage cascade triages columns by difficulty: 46.7% of columns (77/165) were resolved at Stage 1 at negligible cost (<0.001s per column, mean ≈ 55μs), while the remaining 88 (53.3%) advanced to Stages 2/3, with 87/88 terminating at Stage 3. Stage 2 carried the highest per-query latency (mean 0.39s) and dominated cumulative compute (29.7s of 35.7s total wall-clock), while Stage 3 added only 0.08s per query on average for 736-way semantic search. This concentration of compute at Stage 2 reflects the cost of pairwise alias-and-value comparison; Stage 3’s amortized FAISS search is comparatively cheap. End-to-end pipeline time ranged from 0.52s (Krug: 5 columns) to 12.7s (Cao: 29 columns). Total Stage 3 time scaled approximately linearly with the number of columns reaching that stage (Pearson r = 0.868), consistent with the expected linear cost of FAISS search; the full pipeline runtime showed a similar pattern (Pearson r = 0.797) (Figure 4A).

We profiled *OntologyMapper* across four EFO benchmark datasets spanning a ~10-fold range of query sizes, all mapped against a 33,230-term EFO corpus (Figure 4B and C). Cold (first-time) runs were dominated by initialization, taking ~1,200s (~20min) per benchmark regardless of query count, because the corpus pass (embedding 33,230 terms, ~7.5 min) and the synonym pass (embedding ~142,645 texts, ~11min). This cost is incurred only once per *(model, corpus-content)* pair and is amortized across subsequent runs through the warm cache (Methods). On subsequent warm runs, the initialization cost drops to under 0.4s, reducing the total runtime from average ~21.5min to ~22s per benchmark (Supplementary Table 7): Biomappings-EFO was fastest (4.45s) because 72.5% of queries were resolved at Stage 1. OLS-EFO-full was the slowest (43.2s), reflecting its query volume, combined with k=5 FAISS search and alias rerank, on a ~36% non-exact residual. For comparison, *text2term* ran at a consistent ~15s per benchmark from local corpus, regardless of query count (Supplementary Table 8).

### Contamination-controlled evaluation reveals memorization as a confounder in LLM-only schema matching

On the GDC schema-matching benchmark, five frontier LLMs (Claude Haiku 4.5, Sonnet 4.5, and Opus 4.5; Gemini 2.5 Flash and Gemini 2.5 Pro) reached 83.0–89.7% Top-1 accuracy, exceeding *SchemaMapper* with a corresponding alias dictionary by +10.3 to +23.6 pp (matched-model paired comparison; Wilcoxon p < 1 × 10^−5^; Supplementary Table 10). However, 131 of 165 queries (79.4%) were resolved correctly by all five models simultaneously. Such cross-family agreement is uncommon under independent reasoning and is suggestive of a shared exposure to GDC documentation in pretraining data rather than transferable matching capability^32,33^.

**Figure 5.**
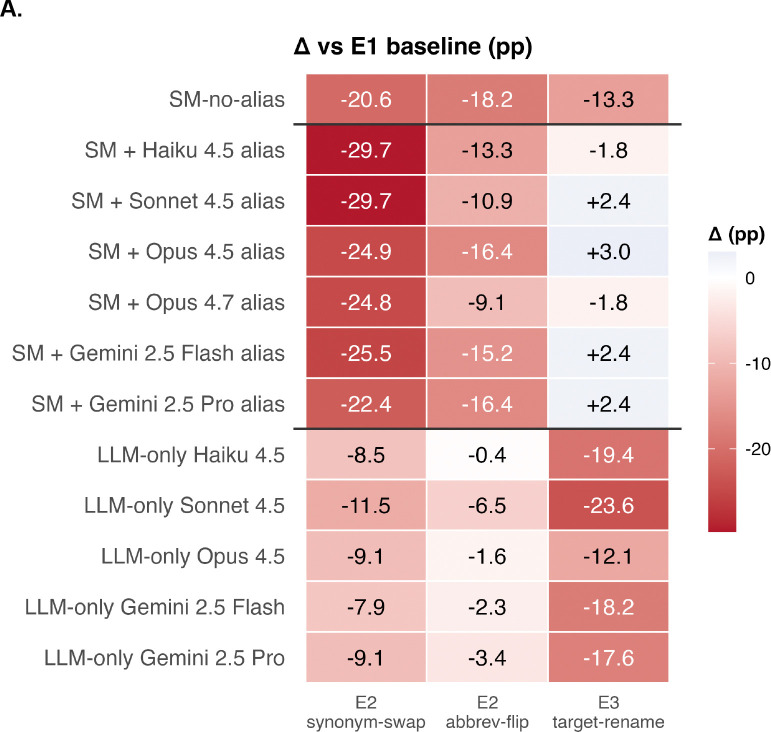

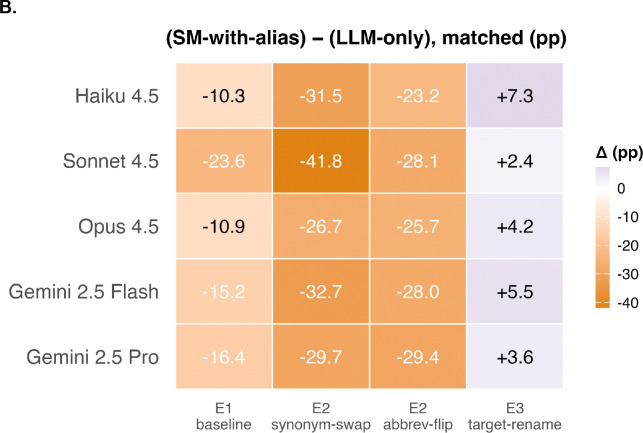
LLM-contamination on schema mapping **(A)** Per-configuration change in Top-1 accuracy under each of the three perturbation conditions, computed as ‘*condition − E1-baseline*’ (Δ). E2 comprises two source-side variants reported separately: synonym-swap and abbreviation-flip. Rows are the 12 configurations grouped into three families separated by horizontal lines: SM with no alias (*SM-no-alias*), SM augmented with LLM-generated aliases (six variants), and LLM-only baselines (five variants; Claude Opus 4.7 was excluded because it does not accept temperature=0). Cell color encodes the signed Δ (red = accuracy loss, blue = accuracy gain). **(B)** Difference in Top-1 accuracy between SM with LLM-alias dictionaries and the LLM-only baseline using the *same* underlying model, computed as ‘*SM-with-alias − LLM-only*’, shown for each of the four conditions (E1 baseline plus three perturbations). Rows are the five matched-model pairs; Opus 4.7 has no LLM-only counterpart in the table and is omitted. Cell color encodes the signed gap (orange = *SM-with-alias* is worse than the LLM-only baseline, purple = *SM-with-alias* is better). When the GDC target labels are renamed (E3), all five *SM-with-alias* configurations overtake their LLM-only counterparts. SM = *SchemaMapper*; cell text reports values as ±*pp*.

Direct memorization probes (E4 with probes P1-P3, defined in Supplementary Methods) test for memorized GDC structure independent of the matching task. (Supplementary Table 11). Three out of five frontier models recovered 80–100% of GDC identifiers from zero-schema context (P2), with matched performance across the Anthropic and Google model families, ruling out a single-lab data-curation artifact; near-zero exact recovery on a positional completion probe (P3: Gemini 2.5 Pro 7%, all others 0%) indicates that this knowledge is indexed by clinical concept rather than stored as a verbatim copy of the 736-column list. For the GDC benchmark test, target-vocabulary rewriting (E3) erased the LLM-only advantage (Supplementary Methods and Supplementary Table 12). All 736 GDC target identifiers were renamed to semantically equivalent non-GDC strings; this collapsed LLM-only Top-1 by 12.1–23.6 pp while *SchemaMapper* was essentially unchanged (−1.8 to +3.0 pp) (Supplementary Table 12). Critically, the paired LLM-only vs. *SchemaMapper* gap collapsed from −10.3 pp at baseline to +7.3 pp under target rename for Haiku 4.5, and from −23.6 pp to +2.4 pp for Sonnet 4.5; across all five matched pairs, *SchemaMapper* paired with an LLM-generated alias dictionary now outperforms the matched LLM-only counterpart by +2.4 to +7.3 pp (Figure 5).

These contamination-controlled evaluations provide direct evidence that GDC concept-to-identifier knowledge is present in both pretraining corpora and confirm the mechanism inferred from the E3 target-rename collapse. LLM-only retained one genuine advantage: robustness to source-column paraphrase, where LLM performance degrades more gracefully than *SchemaMapper*'s retrieval (Supplementary Results).

#### *OntologyMapper* outperforms LLM-only baselines where memorization is not a shortcut.

We evaluated LLM-only performance on the most challenging EFO benchmark datasets, UKBB-EFO. Because the 33,230-term merged corpus exceeds Claude Haiku 4.5's input token limit, this evaluation used a 17,638-term EFO-native corpus; *OntologyMapper* was re-run on the same 17,638-term corpus for a matched comparison. The LLM-only baseline fell ~19–28 pp short of *OntologyMapper* under both zero-shot and open-book prompting (Supplementary Table 13). Closed-book zero-shot prompting reached 54.9% Top-1 (MRR = 0.584) with a 34.5% hallucination rate; injecting the full 17,638-label EFO corpus as a cached system block lifted accuracy to 63.7% Top-1 (MRR = 0.676) and reduced hallucination to 6.0%, but still underperformed *OntologyMapper* (83.0% Top-1, MRR = 0.887; 17,638-term corpus) at a cost of ~$15 (USD) per benchmark run (input: ~322k cache-write + ~143.2M cache-read + ~35k uncached; output: ~129k tokens, totaling ~$15.40 (USD) at Claude Haiku 4.5 (claude-haiku-4–5-20251001) standard real-time API rates as of 2026–04-20 (cache-read alone accounts for ~$14.32)). Per-query agreement analysis between *OntologyMapper* and open-book LLM-only showed *OntologyMapper* was uniquely correct on 24.4% of queries and LLM-only was uniquely correct on only 5.2%, with the remainder either both correct (58.8%) or both wrong (11.6%), indicating *OntologyMapper* encompasses most of the LLM’s capability while contributing substantial unique coverage .

## Discussion

### Reported accuracies of LLM-based metadata harmonization on public biomedical benchmarks can substantially overstate transferable capability.

Because this pattern is observed across two independent pretraining corpora (Claude and Gemini), it is unlikely to be a single-lab curation artifact and instead points to widespread exposure of public schema documentation in pretraining data. The implication extends well beyond *MetaHarmonizer*: any LLM-augmented bioinformatics evaluation built on widely-documented public reference resources (e.g., GDC, GTEx variable dictionaries, TCGA harmonized schemas) should be interpreted as an upper bound that may not transfer to novel or proprietary schemas absent from pretraining. The contamination battery (Supplementary Methods) is task-agnostic and can be applied wherever an LLM baseline is reported against a public benchmark. This kind of probing may be a useful addition to LLM-augmented benchmarks, in the same way that train-test leakage checks are now standard for supervised learning evaluations.

This finding reframes the methodological question for biomedical metadata harmonization; not whether LLMs can match documented schemas they may have memorized, but how to build harmonization systems that remain reliable on novel schemas, in offline settings, and under the FAIR-compliant data infrastructure. *MetaHarmonizer* is one response to that question: a multi-staged retrieval architecture that uses LLMs only as bounded components within a deterministic and auditable pipeline, achieving competitive accuracy with grounded outputs, calibrated confidence, and stable performance under contamination probes.

### LLMs as components rather than as solutions.

*SchemaMapper* uses LLMs for one targeted role – alias dictionary generation as one-time preprocessing – while grounding all outputs in a pre-defined schema. Its performance was stable across the contamination probing conditions because the core matching algorithm depends on embedding similarity to target column names rather than memorized schema knowledge. This robustness is from the architectural choice: contamination affects the alias dictionary's coverage but not the deterministic matching that follows.

### Aliasing matters, but the choice of the LLM model for alias generation mostly does not.

A prevalent assumption in LLM-augmented bioinformatics is that a more capable model uniformly improves output quality. We find a weaker pattern for embedding-friendly synonym generation: six top-tier proprietary models generated alias dictionaries whose downstream *SchemaMapper* performance was statistically indistinguishable on Top-1 accuracy after multiple-comparison correction, despite spanning roughly a 50-fold range in API cost. The failure mode of larger models is not incorrect aliases but too many correct-but-off-task ones, such as pipeline tool names and value-like strings that are plausible surface forms yet poor semantic neighbors of the actual queries. Because *SchemaMapper* Stage 3 ranks aliases by cosine similarity, these embedding-unfriendly aliases pull unrelated queries toward the wrong field or dilute rank by occupying independent embedding slots. The practical consequence is that Haiku-4.5 is the Pareto-optimal default for *SchemaMapper* alias dictionary generation (Supplementary Figure 1 and Supplementary Table 15). The implication generalizes beyond *MetaHarmonizer*: in any retrieval pipeline that consumes LLM-generated text via dense embeddings, model selection should be aligned with the downstream consumer, not general capability benchmarks. This suggests that within the equivalence set, users can pick on cost, latency, vendor, or compliance option without compromising accuracy.

Beyond accuracy, the LLM-generated alias dictionary requires only target column names as input, making adaptation to new schemas a matter of providing column names and, optionally, modified prompt templates. Direct cross-schema validation remains future work, but the *source-agnostic generation procedure* provides easy adaptation to new domains.

### *SchemaMapper* recovers more plausible candidates for human review.

With the LLM-generated alias dictionary, *SchemaMapper* achieved the highest per-query Recall@GT among the tested, including Magneto’s fine-tuned bipartite variant. This advantage reflects a benefit of our multi-stage cascade design: it draws candidates from structurally different matching strategies, each with distinct biases, and naturally surfaces multiple acceptable targets within its candidate list. In real-world schema harmonization where one-to-many mappings are common, curating a diverse high-quality candidate set for human review is often more valuable than rank-1 precision alone. Magneto's fine-tuned LLM-reranker on MRR is statistically indistinguishable from *SchemaMapper*+Opus-4.5-alias, and the bipartite reranker configurations are beaten by *SchemaMapper*+Opus-4.5-alias and statistically indistinguishable from *SchemaMapper*+Haiku-4.5-alias. Thus, the two systems can occupy complementary points on the precision–recall frontier: auto-fill workflows benefit from Magneto's LLM-reranker, while human-in-the-loop review benefits from *SchemaMapper*.

### *OntologyMapper*'s accuracy advantage stems from multi-stage semantic matching.

*OntologyMapper* achieved Top-1 accuracy of 77.9–95.5% and MRR of 0.83–0.97 across four EFO benchmarks (on a 33,230-term corpus, micro-average), consistently outperforming *text2term*. The performance gap derives primarily from semantic matching stages; SapBERT was pre-trained on millions of UMLS synonym pairs and place semantically equivalent terms close together regardless of lexical overlap, while *text2term*'s TF-IDF backbone is sensitive to surface-form variation. Performance variation across benchmarks is interpretable from source-term properties: UKBB-EFO yielded the lowest accuracy because UK Biobank phenotype descriptions are often informal or use lay terminology (e.g., "blood clot in the lung" vs. "pulmonary embolism"), reducing Stage 1 exact-match resolution. The OLS-EFO disease subset outperformed the full benchmark because disease ontology terms have more extensive synonym sets and more consistent naming conventions. The two tools occupy complementary positions: *text2term* as a lightweight, broadly accessible solution well-suited to lexically aligned tasks; *OntologyMapper* as a higher-accuracy option for semantically challenging mappings, at the cost of transformer embeddings.

### Cascade design makes the system tractable.

Each module's "easy-first" design resolves simple cases before invoking expensive methods. *OntologyMapper*'s Stage 1 provides a high-confidence, zero-error pathway for terms with direct lexical matches, resolving 32.4–72.5% of queries before embedding-based stages are invoked. Stage 2 handles the bulk of semantically complex matching, while Stage 2.5 provides a safety net through expanded synonym search. Beyond accuracy, this organization yields predictable runtime: *SchemaMapper* remains under 30 sec even for the largest CPTAC studies, and *OntologyMapper*'s per-query marginal cost of 0.006s suggests the pipeline accommodates tens of thousands of terms without bottlenecks. *OntologyMapper*'s cold/warm asymmetry reflects one-time embedding passes cached per corpus, a cost model well-suited to typical usage where a laboratory or consortium maps multiple studies against the same target ontology over time.

### Calibrated confidence enables practical triage.

Both modules produce confidence scores with strong separation between correct and incorrect predictions, and this separation holds consistently across studies and benchmarks. In production workflows where curators must validate predictions, well-calibrated confidence is often more useful than marginal accuracy gains: predictions above a chosen threshold can be auto-accepted while those below are flagged for review. This thresholding capability is a direct consequence of staged retrieval – each stage's score reflects a specific matching mechanism with bounded failure modes – and is harder to obtain from end-to-end approaches that produce a single output.

### Limitations.

Several limitations should be acknowledged. The contamination battery establishes that LLM-only advantages on the GDC benchmark disappear upon target renaming, but the magnitude and form of contamination effects on other public schemas have not been characterized. Benchmarks span two domains – cancer genomics and disease ontology – and broader coverage would strengthen claims of generalizability. Each module was evaluated against a single primary comparator; the broader landscape of schema matchers (e.g., Valentine^31^, COMA^34^) and ontology mappers (e.g., ZOOMA^35^) was not exhaustively benchmarked. Finally, while *MetaHarmonizer* is designed to be domain-extensible (i.e., target schemas and ontology corpora are user-supplied rather than hard-coded), empirical validation outside the benchmarked domains remains future work.

### Future directions.

Applying the contamination protocol systematically across other LLM-augmented bioinformatics benchmarks built on public reference resources is a natural next step. For *MetaHarmonizer* specifically, applying the framework to additional repositories and data types would both extend its practical utility and stress-test its coverage on more heterogeneous real-world inputs. More broadly, harmonization is one component of a larger curation workflow, and integration with complementary tools (e.g., those that extract structured metadata from unstructured sources) could position *MetaHarmonizer* within an end-to-end metadata pipeline. Each of these directions builds on, rather than reorganizes, the *MetaHarmonizer* foundation established here.

## Conclusions

*MetaHarmonizer* demonstrates that staged retrieval with bounded LLM components is a practical architecture for biomedical metadata harmonization at scale. *MetaHarmonizer* is locally deployable and deterministic, and its calibrated confidence scores translate accuracy into actionable triage. As biomedical data repositories continue to expand and cross-study analyses become routine, harmonization frameworks will need to scale not only computationally but also in the trust they earn from curators and downstream users. *MetaHarmonizer* is built for both.

## Supplementary Material

Supplement 1

## Figures and Tables

**Figure 1.**
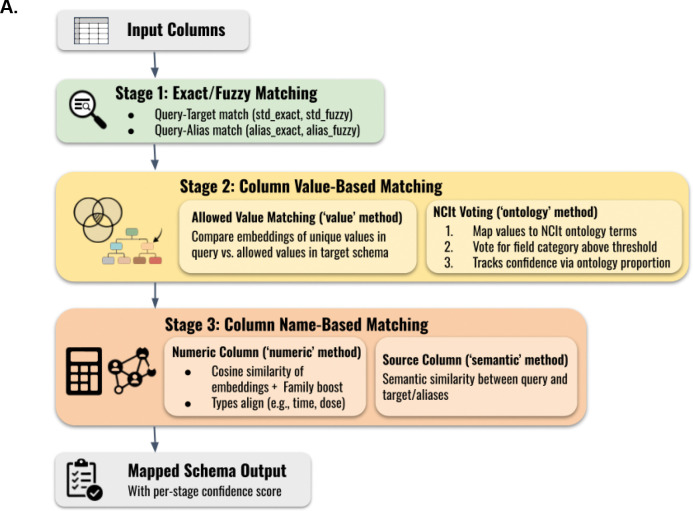

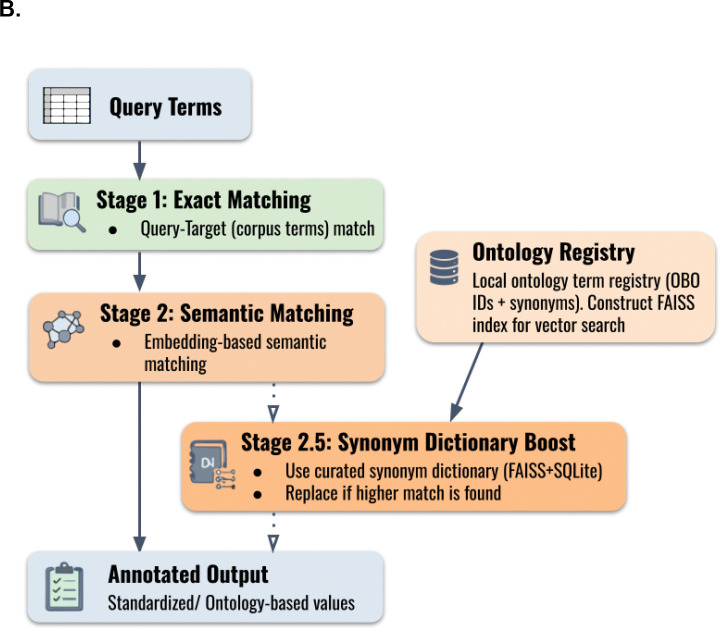
Overview of *MetaHarmonizer* *MetaHarmonizer* performs metadata harmonization at two levels: attribute-level via *SchemaMapper* and value-level via *OntologyMapper*. Each module cascades queries through stages, applying low-cost lexical methods first and reserving embedding-based methods for unresolved queries. **(A)** *SchemaMapper* aligns input column names to a target schema in three sequential stages. Stage 1 (green) performs exact and fuzzy matching against target names and their aliases. Unresolved queries advance to Stage 2 (yellow), which uses column-content evidence: the 'value' method compares embeddings of unique values across source and target columns, while the 'ontology' method maps values to ontology terms and votes for a target field when >20% match, tracking confidence via the matched proportion. Remaining queries enter Stage 3 (orange), which compares column-name embeddings: the 'numeric' method applies a family-aware boost when types align (e.g., time, dose), and the 'semantic' method handles ambiguous headers via target/alias similarity. Each output is annotated with its resolving stage and a per-stage confidence score. **(B)** *OntologyMapper* grounds free-text values to ontology terms in two primary stages, plus a dictionary-boost step. Stage 1 (green) performs exact matching against the corpus terms. If there is no confident match, queries advance to Stage 2 (orange), which retrieves candidates based on cosine similarity between the query and target embeddings, using a FAISS index over a local ontology registry. Low-confidence Stage 2 outputs trigger Stage 2.5, which queries a curated synonym dictionary (FAISS + SQLite) and replaces the candidate if a higher-scoring match is found. The outputs are standardized, ontology-grounded values.

**Table 1.** Benchmark comparison of *SchemaMapper* and *Magneto* on the GDC datasets

Method	Training	MRR (macro)	Top-1 (%)	Top-5 (%)	Recall@GT (per-query)	Recall@GT (global)
SM (no alias)	Zero-shot	0.521	47	61.5	0.425	
SM + Opus 4.5 alias	Zero-shot	0.811	77.1	87	0.685	—
SM + Haiku 4.5 alias	Zero-shot	0.763	71.6	83.2	0.65	—
SM + Gemini 2.5 Pro alias	Zero-shot	0.76	72.8	80.9	0.659	—
Magneto-zs-bp †	Zero-shot	0.731	62.1	86.5	0.537	0.373
Magneto-ft-bp †	Fine-tuned	0.757	68.4	85.3	0.601	0.433
Magneto-zs-llm *	Zero-shot	0.808	—	—	—	0.43
Magneto-ft-llm *	Fine-tuned	0.846	—	—	—	0.537

Ten CPTAC data tables were mapped to the 736-column GDC target schema, and performance was evaluated using ground-truth matches for 165 source columns created through expert curation. We compared the no-alias SM baseline, SM with three LLM-generated alias dictionaries (the best vendor-diverse option; see Supplementary Results), and four Magneto variants. Recall@GT is reported under two non-interchangeable definitions, *per-query* and *global*, which differ in whether they penalize cross-query score miscalibration; the two columns should not be read as adjacent values (see Supplementary Methods for definitions and the conditions under which each is computable). *SchemaMapper* produces a sparse candidate list and cannot support the global Recall@GT definition, and Magneto-llm rows do not include the per-query rank distributions needed to compute Top-1 and Top-5. All cells are *macro-averaged* across studies (per-study metric first, then unweighted mean over the ten studies).

Asterisked (*) Magneto LLM variants are paper-reported (Liu et al.^1^ Table 6, MPNet rows), not reproduced locally.

Daggered (†) Magneto bipartite variants were reproduced locally, matching the source paper within ±0.005 on both MRR and global Recall@GT.

“—” represents a metric that is unavailable in the source data. SM = *SchemaMapper*; zs = zero-shot; ft = fine-tuned; bp = bipartite-reranker; llm = LLM-reranker.

**Table 2.** Benchmark comparison of *OntologyMapper* and *text2term* across four EFO mapping datasets

Benchmark	Tool	n	Top-1 (%)	Top-3 (%)	Top-5 (%)	MRR (micro)
UKBB-EFO	OM	888	77.9	87	89.4	0.826
	t2t	888	71.6	81	83.3	0.765
Biomappings-EFO	OM	795	95.5	98.2	98.7	0.969
	t2t	795	79.1	89.7	93.2	0.848
OLS-EFO (disease)	OM	5,770	95.2	97.3	97.9	0.963
	t2t	5,824	93	97.2	98	0.952
OLS-EFO (full)	OM	7,377	89.1	91.5	92.1	0.903
	t2t	7,504	79.2	83.5	84.2	0.813

Ontology mapping performance was evaluated on four ground-truth benchmarks of increasing scale and heterogeneity. The target corpus includes the 12-ontology EFO selection (33,230 terms for OM and 33,659 terms for t2t). For each benchmark, *n* indicates the number of query terms with at least one ground-truth target. Small differences in *n* between tools reflect tool-specific handling of queries that yield no candidates within the configured search space. *OntologyMapper* outperforms *text2term* across all four benchmarks and metrics, with the largest absolute gains observed on Biomappings-EFO and the smallest on OLS-EFO (disease), where both tools approach ceiling performance. We used the Top-5 retrievals and the micro-average for this evaluation. OM = *OntologyMapper*; t2t = *text2term*.

## Data Availability

Benchmark datasets used in this study are available from the corresponding source repositories: https://github.com/VIDA-NYU/magneto-matcher for GDC schema mapping, and https://github.com/rsgoncalves/text2term-evaluation for EFO ontology mapping.

## Code Availability

The *MetaHarmonizer* Python package is available in this GitHub repository: https://github.com/shbrief/MetaHarmonizer .
