# Supplementary material for: MetaHarmonizer: robust biomedical metadata harmonization and a contamination control for inflated LLM performance on public benchmarks
Source: bioRxiv. 2026 Jun 17:2026.06.13.732088. Preprint. [Version 1] doi: 10.64898/2026.06.13.732088 (PMC13308024; doi:10.64898/2026.06.13.732088)
Supplement: Supplement 1 [file NIHPP2026.06.13.732088v1-supplement-1.pdf]

bioRxiv preprint doi: https://doi.org/10.64898/2026.06.13.732088; this version posted June 17, 2026. The copyright holder for this preprint (which was not certified by peer review) is the author/funder, who has granted bioRxiv a license to display the preprint in perpetuity. It is made available under a CC-BY 4.0 International license.

# Supplementary Methods

## Schema-matching evaluation: metric definitions and aggregation choices

Cross-method comparison on the GDC schema-matching benchmark involves several closely related but non-equivalent metrics, two distinct aggregation schemes, and one cascade-specific implementation property that affects reported numbers. We document each below.

***Per-query metrics.*** For each source column *c*, each method returns a ranked list of candidate target columns $\widehat{T}_c = (t_1, t_2, ..., t_k)$, truncated at *K* predictions. Let G*c* denote the set of ground-truth targets for *c* with $|G_c| = n_c$. We compute three per-query metrics from this ranked list.

- *Mean Reciprocal Rank (MRR).* Following Liu et al.[1], the per-column reciprocal rank is $RR(c) = 1/rank(c)$, where $rank(c)$ is the position of the first correct target in $\widehat{T}_c$. If no correct target appears within Top-k, $RR(c) = 0$. To match the cutoff used in the Magneto evaluation (Magneto-bp variants use the default `topk = 20`), we fix $K = 20$ for all *SchemaMapper* runs subjected to direct comparison against Magneto outputs.
- *Top-k accuracy.* $Top_k(c) = 1$ if any element $G_c$ appears in the first *k* predictions, and 0 otherwise. We report k = 1-5.
- *Per-query Recall@GT.* Following the per-query formulation in Liu et al.[1], $Recall@GT_{pq}(c) = |\widehat{T}_c[1{:}min(n_c, 5)] \cap G_c|/n_c$. The truncation $min(n_c, 5)$ follows the released code and prevents the metric from rewarding methods solely for returning longer candidate lists.

***Global Recall@GT.*** Whereas Top-k and MRR depend only on the ordering of candidates within each query and therefore have no analogous "global" form, Recall@GT can be computed against a study-wide ranked list of (query, target) pairs scored across all queries. The published Magneto results report a *global* Recall@GT, computed by flattening each study's full source–target similarity matrix (up to 165 × 736 for this benchmark) into a list of $(c, t, s_{c,t})$ triples ranked by score $s_{c,t}$, retaining the top $|G| = \sum_c n_c$ triples, and reporting the fraction that are ground-truth pairs. Unlike per-query Recall@GT, the global formulation requires that every (*c*, *t*) pair receive a comparable score, thereby penalizing cross-query score miscalibration: a method that ranks correct matches highly within each query but inconsistently across queries can score well per-query yet poorly globally.

*SchemaMapper* produces a sparse output by design: it roughly scores 5–20 candidates per query rather than the full target schema. Global Recall@GT is therefore not computable for

bioRxiv preprint doi: https://doi.org/10.64898/2026.06.13.732088; this version posted June 17, 2026. The copyright holder for this preprint (which was not certified by peer review) is the author/funder, who has granted bioRxiv a license to display the preprint in perpetuity. It is made available under a CC-BY 4.0 International license.

*SchemaMapper* without modifying the architecture to score every (*c*, *t*) pair. We restrict *SchemaMapper* to per-query Recall@GT and report both formulations side by side for the Magneto-bp variants where both are available, leaving cells blank where the underlying scoring data does not support a given metric (**Table 1**).

***Macro vs. micro aggregation.*** The published Magneto results are reported as macro averages, so we adopt macro averaging throughout the cross-method comparison in Table 1 to match the source paper's aggregation convention. With n = 165 source columns distributed unevenly across 10 studies (n = 5-29), the choice of aggregation matters. Micro and macro averages of Top-k for the same *SchemaMapper* + Haiku-4.5-alias run differed by 1.73 pp at Top-1 and 0.44 pp at Top-5; the corresponding gap at MRR was the difference between micro (0.771) and macro (0.763) at Top-5. Mixing aggregation schemes across methods would introduce a systematic bias of comparable magnitude to the smallest method differences we report. The applicability of each aggregation scheme to each metric is summarized in **Supplementary Table 14**.

***Cross-method comparability.*** MRR is computed identically for all four Magneto variants and both *SchemaMapper* configurations (Top-20 ranked list, macro-averaged across the 10 studies), and is therefore directly comparable across all six rows in Table 1. Top-1 and Top-5 accuracy are computed identically for *SchemaMapper* and the two Magneto-bp variants we reproduced locally; the Magneto-llm rows are not reported at the rank-distribution level needed to recompute these values without rerunning the proprietary LLM reranker. Per-query Recall@GT is comparable across the four reproducible rows (*SchemaMapper* variants and Magneto-bp variants), and global Recall@GT is comparable across the four Magneto rows (paper-reported and locally reproduced). We populate each cell only when the underlying scoring data support the metric and the formulation is consistent with the comparison group, and leave cells blank rather than substituting numerically incomparable values. Reproduction fidelity for the Magneto-bp variants is reported in the Table 1 caption.

***Cascade-dependent rank behavior.*** The *SchemaMapper* cascade decides when to advance between stages based on the Top-1 confidence and merges results from each stage using Top-k-dependent rules. As a result, requesting more candidates does not simply extend the same ranked list – it can change which predictions appear in the Top-k, and the Top-5 of a k = 20 run is not in general identical to the Top-5 of a k = 5 run. To eliminate this confound from the cross-method comparison, all *SchemaMapper* numbers reported against Magneto are computed from a single k = 20 run, matching Magneto's `topk = 20` setting.

***Confidence calibration.*** Confidence calibration metrics (AUC, Cohen's *d*, Wilcoxon rank-sum) are computed directly over a pool of (prediction, correctness, confidence) records aggregated across all studies, rather than by aggregating per-query values. Because they have no per-query form, the macro/micro distinction does not apply (**Supplementary Table 14**). Within-study computation is degenerate for the smallest studies (e.g., Krug, *n* = 5), where the positive or negative class may be empty, and AUC is undefined; even when both classes are populated, sample sizes below ~15 yield estimates with prohibitively wide confidence intervals. Pooling across studies avoids both failure modes and is the formulation we report.

bioRxiv preprint doi: https://doi.org/10.64898/2026.06.13.732088; this version posted June 17, 2026. The copyright holder for this preprint (which was not certified by peer review) is the author/funder, who has granted bioRxiv a license to display the preprint in perpetuity. It is made available under a CC-BY 4.0 International license.

## Denominator conventions across analyses

The four *OntologyMapper* benchmark inputs are not uniform in row count: UKBB-EFO and Biomappings-EFO have one row per query (888 and 795, respectively), while OLS-EFO (disease) and OLS-EFO (full) contain multi-target rows – the same source string appears multiple times with different ground-truth EFO labels – yielding 5,824 and 7,504 input rows, only 5,770 and 7,377 unique query strings, and 5,804 and 7,445 output rows. We adopt three different denominators across the analyses below, each matching the unit the corresponding metric is intended to summarize.

***Runtime analysis*** uses the **input benchmark row count** (888 / 795 / 5,824 / 7,504) because that is the workload each tool was handed. This measure answers "how long does the workflow take", instead of "how fast is the matching algorithm itself", which can be answered by using unique-query as the denominator. Both *OntologyMapper* and *text2term* internally deduplicate queries before invoking the matching backend, but the user-observable runtime (and the resulting output row count, after both tools expand results back to benchmark scale) is keyed to the input. The input row count, therefore, reflects the work actually executed and yields directly comparable per-query throughput across tools.

***Confidence-calibration analysis*** (mean confidence by correctness, Cohen's d, AUC for correct-vs-incorrect separation) uses the **module output row count** (890 / 795 / 5,804 / 7,445 for *OntologyMapper*). Each emitted row is an independent prediction with its own Top-1 score; the calibration question is row-level ("do correct predictions score higher than incorrect ones?"), so collapsing rows would discard signals. *OntologyMapper* occasionally emits byte-identical duplicate rows for the same query (2 in UKBB; 34 in OLS-disease; 68 in OLS-full); these are retained because they represent independent module evaluations.

***Top-K accuracy and MRR*** use the **unique-query count** (888 / 795 / 5,770 / 7,377 for *OntologyMapper*; 888 / 795 / 5,824 / 7,504 for *text2term*). Accuracy answers a query-level question, "among the distinct mappings we wanted, what fraction landed in the Top-K?". *OntologyMapper* deduplicates queries before Stage 2, so multi-target benchmark rows that don't get resolved at Stage 1 collapse to a single matcher evaluation; *text2term* deduplicates before its TF-IDF call and then left-joins results back onto the full benchmark, so its denominator matches the raw benchmark row count. We report these conventions each tool naturally produces rather than forcing a common denominator, since either choice introduces an artificial bias. Query-count differences between tools (0–127 rows per benchmark) therefore reflect divergent handling of benchmark rows that share a query string but map to different targets, rather than differences in evaluation rigor. For all three analyses, ranking is computed case-insensitively, matching *text2term*'s built-in evaluation logic.

## Statistical analysis for LLM-generated alias comparison

All comparisons are paired on the same 165 queries. For Top-k accuracy, we used McNemar's exact two-sided test on the contingency of (rank ≤ k) indicators between method pairs. For MRR and Recall@GT, we used the paired Wilcoxon signed-rank test on per-query metric values. For

bioRxiv preprint doi: https://doi.org/10.64898/2026.06.13.732088; this version posted June 17, 2026. The copyright holder for this preprint (which was not certified by peer review) is the author/funder, who has granted bioRxiv a license to display the preprint in perpetuity. It is made available under a CC-BY 4.0 International license.

each metric, we performed 28 pairwise comparisons across the eight methods. p-values were corrected for multiple comparisons by Holm–Bonferroni within each metric (m = 28). Where a Tier-A subset analysis is reported, Holm–Bonferroni was applied to the 15 within-Tier-A pairs (m = 15) as a planned-subset correction. Paired bootstrap confidence intervals on per-method MRR/Top-1/Top-5 and on the pairwise ΔMRR / ΔTop-k vs. Opus 4.5 were computed from B = 10,000 resamples (`set.seed(42)`); each bootstrap resample used the same query indices for every method to preserve pairing. A method's CI on *'Δ vs. Opus 4.5'* was classified as "statistically equivalent to Opus 4.5" when it crossed zero, and as "significantly worse" otherwise (no method had a CI lying entirely right of zero).

## LLM-only baseline for schema mapping

The prompting configuration was driven by a common task-specific system instruction for schema matching (*"You are a biomedical data expert. Your task is to match a source metadata column to the most appropriate target column from a standardized schema. Return ONLY a JSON array; no prose, no markdown."*) and a shared user template that listed all target columns and requested the Top-5 matches as a JSON array of {target, confidence} objects. The returned target was checked for membership in the target schema, and predictions outside the schema were labeled as hallucinations. Top-k accuracy (k = 1,3,5), MRR, and Recall@GT were computed; 95% confidence intervals on Top-k accuracy were bootstrap replicates (n = 1,000). Paired comparisons between configurations use the Wilcoxon signed-rank test on per-query rank differences (one-sided). For Top-1 correctness, we additionally report discordant-pair (McNemar-style) counts with an exact binomial test against p = 0.5. Five frontier models spanning two independent pretraining corpora were evaluated on GDC: Claude Haiku 4.5, Claude Sonnet 4.5, and Claude Opus 4.5 (Anthropic), as well as Gemini 2.5 Pro and Gemini 2.5 Flash (Google). All calls used `temperature = 0`, where the provider permits. Opus 4.7 was used for *SchemaMapper* alias generation but was excluded from LLM-only because it does not accept `temperature = 0`. Requests were issued sequentially at 1s intervals with exponential backoff retries. All responses were cached on disk to make re-runs deterministic.

## LLM-only baseline for ontology mapping

An LLM-only baseline was evaluated on the 888-query UKBB-EFO benchmark using the EFO corpus (17,638 terms from EFO v3.62.0). Unlike the corpus used for benchmarking against *text2term*, it includes only EFO-native classes, and imported, non-EFO classes (e.g., from HP, MONDO, etc.) are excluded. We used this smaller corpus because the 33,230-term EFO corpus exceeds Haiku 4.5's allowed input token size. Two prompting configurations were compared: 1) *zero_shot* - a role instruction only with the user message containing only the query phrase and a request for the Top-5 EFO labels; 2) *open_book_full* - the full EFO corpus injected as a cached system block with Anthropic's ephemeral prompt caching, with a "verbatim from the list" constraint in the user message. The ~161k-token corpus block is billed once upon first creation and then read at ~10% of the base input rate for the remainder of the ephemeral TTL (Time to Live), making the full 888-query pass affordable. Each returned label was checked against the 16,218 unique EFO labels (17,638 terms without 1,419 obsolete terms and 1 duplicated term). Predictions absent from it were labeled as hallucinations. Accuracy at Top-k and bootstrap 95%

bioRxiv preprint doi: https://doi.org/10.64898/2026.06.13.732088; this version posted June 17, 2026. The copyright holder for this preprint (which was not certified by peer review) is the author/funder, who has granted bioRxiv a license to display the preprint in perpetuity. It is made available under a CC-BY 4.0 International license.

CIs were computed in the same way as in the main benchmarking. Claude Haiku 4.5 was used with `temperature = 0`, `max_tokens = 1024`, sequential requests at 1s intervals, and up to 5 exponential-backoff retries for transient errors. All responses were cached on disk.

## LLM contamination control for schema mapping

Because the GDC data model and CPTAC harmonization mappings are openly documented and present in common web-scale pretraining corpora, strong LLM-only performance on the GDC benchmark may reflect memorization of target-schema identifiers rather than transferable schema-matching capability. To quantify the contribution of memorization, we applied a three-part battery (E2, E3, E4) to each configuration on the GDC benchmark in addition to the baseline (E1). We ran identical perturbations through three configurations: 1) *SchemaMapper* without an alias dictionary, a pure embedding-plus-retrieval pipeline referred to below as '*SM-no-alias*', 2) *SchemaMapper* with each of six LLM-generated alias dictionaries (*SM+alias*; Haiku 4.5, Sonnet 4.5, Opus 4.5, Opus 4.7, Gemini 2.5 Flash, Gemini 2.5 Pro), and 3) *LLM-only*. For LLM-only, we evaluated the same five frontier models defined in the LLM-only baseline for schema mapping (**Supplementary Methods**): Opus 4.7 was excluded because it rejects `temperature=0` and would not have produced comparable deterministic outputs.

***Source-side perturbations (E2)*** rewrite the source column names without changing ground truth. Synonym-swap uses Claude Sonnet 4.5 (`temperature = 0`) to generate a snake_case paraphrase, rejecting and regenerating any output that matches a GDC target name exactly (up to two retries). Abbreviation-flip expands abbreviated source names to full form and vice versa. A 20% random sample (25 of 124 unique sources) was audited for semantic preservation. Crucially, for the *SM+alias* configurations under E2, the original alias dictionaries were retained unchanged.

***The target-side perturbation (E3)***. All 736 GDC target column identifiers were renamed to semantically equivalent non-GDC-style strings using Sonnet 4.5 (0 empty outputs; 0 collisions with original GDC names; 147 / 736 rows audited); thus, the semantic matching capability is preserved by construction. Ground truth was translated through the rename map, so a "correct" prediction under E3 is the renamed label for the same source concept. For the *SM+alias* configurations, the alias dictionary's `field_name` column was renamed, so a 'correct' answer under E3 is the renamed target. Any perturbation output that exactly matches a GDC target name was rejected (case-insensitive, up to 2 retries) to prevent LLM-only from using memorized mapping. The E3 paired comparison is computed on the subset of unique source columns (n = 124) rather than the full 165-row benchmark, because several source columns recur across CPTAC studies and would otherwise contribute correlated pairs that violate the independence assumption of the paired Wilcoxon test. E1 baseline accuracies are recomputed on the same unique subset, so the comparison is apples-to-apples. Qualitative conclusions are unchanged.

***Direct memorization probes (E4)***. Independent of the schema-matching task, we employed three diagnostic tests (probes, *P*) to test for memorized GDC structure. *P1* asks each model to list, from zero context, all columns of the GDC demographic node and scores the fraction of output lines whose first token is a real GDC column. *P2* asks for the exact GDC identifier for

bioRxiv preprint doi: https://doi.org/10.64898/2026.06.13.732088; this version posted June 17, 2026. The copyright holder for this preprint (which was not certified by peer review) is the author/funder, who has granted bioRxiv a license to display the preprint in perpetuity. It is made available under a CC-BY 4.0 International license.

each of 20 common clinical concepts ("biological sex", "AJCC pathologic tumor stage"), with no schema provided, and reports the exact-match rate. *P3* presents a 12-name slice of the GDC schema, with three names randomly masked as `???`, and asks the model to fill them in (10 slices, seeded); we report the exact recovery rate.

***Duplicate-query handling.*** The 165-mapping benchmark contains 124 unique source column names; 26 source strings recur across multiple CPTAC studies (e.g., `Stage` appears in three studies, `Tumor_Site` in two). These are not redundant evaluations: the gold-standard `ref_match` is defined per source study, so the same column name can resolve to different harmonized GDC targets across studies. All Top-1 and MRR accuracies are reported at the row level over the full n=165 evaluations, preserving per-study labels. Paired comparisons between baseline (E1) and perturbed runs (E2, E3) are matched on the `(query, source_study)` key rather than `query` alone. LLM predictions are deterministic under `temperature=0` and we confirmed that every duplicated source string produced identical Top-1 predictions across its copies in all E1/E2/E3 runs; nonetheless, correctness diverges across copies when the per-study gold differs.

bioRxiv preprint doi: https://doi.org/10.64898/2026.06.13.732088; this version posted June 17, 2026. The copyright holder for this preprint (which was not certified by peer review) is the author/funder, who has granted bioRxiv a license to display the preprint in perpetuity. It is made available under a CC-BY 4.0 International license.

# Supplementary Results

## *SchemaMapper* Alias Dictionary Generation Feasibility and Cost

Of the nine models tested, seven successfully completed the 736-field alias dictionary generation, achieving 100% field coverage; per-model row counts, wall times, and API costs are reported in **Supplementary Table 9**. Gemma3:27b required post-hoc filtering of 185 rows associated with 105 hallucinated field names (1.9% of generated output) before downstream use. Two open-weight models failed outright: Gemma4:26b produced malformed output that could not be parsed, while Qwen3:32b returned *structurally* valid output in which the source-alias column contained the literal letters A–F (the placeholder labels from the few-shot example table) rather than real alias strings, a failure mode sometimes referred to as template capture. Because the prompt was held fixed across all generators for parity, we did not attempt prompt-level rehabilitation of Qwen3:32b.

***Top-tier LLM-generated alias models are mutually interchangeable under family-wise correction; the equivalent set narrows under per-pair confidence intervals.*** Six of the seven alias models — Opus 4.5, Haiku 4.5, Gemini 2.5 Pro, Opus 4.7, Gemini 2.5 Flash, and Sonnet 4.5 (henceforth 'Tier-A') — significantly outperformed the `no-alias` *SchemaMapper* baseline on Top-1 after Holm–Bonferroni correction over the 28 all-pairs McNemar comparisons (Holm-adjusted $p \leq 0.032$). Gemma 3 27B did not (Holm-p = 1.0; raw p = 0.54).

*Family-wise comparison, answering "is any Tier-A pair reliably different from any other?":* Within Tier-A, all 15 within-tier comparisons were mutually non-significant under the same 28-pair family (Holm-adjusted McNemar $p \geq 0.105$ within the Tier-A 15 pair family, $p \geq 0.119$ across all 28 pairs). The same conclusion held for Recall@GT (Holm-adjusted paired Wilcoxon signed-rank $p \geq 0.273$ within Tier-A, $p \geq 0.291$ across all 28 pairs). MRR was the most sensitive continuous metric, identifying two within-Tier-A pairs as significant (Opus 4.5 > Sonnet 4.5, Holm-p = 0.004; Opus 4.5 > Gemini 2.5 Flash, Holm-p = 0.042) and two as borderline (Opus 4.5 > Opus 4.7 and Haiku 4.5 > Sonnet 4.5, Holm-p = 0.052 each). Top-3 and Top-5 each identified one within-Tier-A pair as significant, Opus 4.5 > Opus 4.7 (Top-3 Holm-p = 0.026; Top-5 Holm-p = 0.003). Notably, Opus 4.5 vs. Haiku 4.5 was statistically indistinguishable on every metric (Holm-p = 1.0 across Top-1, Top-3, Top-5, MRR, and Recall@GT).

*Per-pair comparison, answering "can any single model be comparable to Opus 4.5 as a deployment option?":* Per-pair 95% paired-bootstrap CIs on $\Delta$ vs. Opus 4.5 are stricter than the Holm-corrected pairwise test, and the equivalent set narrows as the metric becomes more discriminative (**Supplementary Figure 1A-C**). The ΔMRR CI crosses zero only for Haiku 4.5 and ($\Delta = -0.029$, CI [−0.078, +0.019]) and Gemini 2.5 Pro ($\Delta = -0.041$, CI [−0.091, +0.006]). The ΔTop-1 CI additionally includes Opus 4.7 ($\Delta = -5.5$ pp, CI [−11.5, +0.6 pp]), though this is the most fragile equivalence call, with the upper bound only just crossing zero. At Top-5, the equivalent set collapses to just Opus 4.5 and Haiku 4.5. The ΔRecall@GT CI crosses zero only for Haiku 4.5 and Gemini 2.5 Pro.

bioRxiv preprint doi: https://doi.org/10.64898/2026.06.13.732088; this version posted June 17, 2026. The copyright holder for this preprint (which was not certified by peer review) is the author/funder, who has granted bioRxiv a license to display the preprint in perpetuity. It is made available under a CC-BY 4.0 International license.

***Two Tier-A models are strictly Pareto-dominated.*** Plotting Top-1 / MRR against one-time generation cost revealed the Pareto frontier of cost-efficient alias models (**Supplementary Figure 1D**). Among the API-hosted methods, the frontier (in ascending cost) consists of Gemini 2.5 Flash ($0.49) → Haiku 4.5 ($1.04) → Gemini 2.5 Pro ($2.00) → Opus 4.5 ($21.51). Two Tier-A methods are off-frontier and strictly dominated by the same-vendor alternative: Sonnet 4.5 ($4.45, MRR 0.708) is dominated by Haiku 4.5 ($1.04, MRR 0.771): the same vendor, ≈ 4× more expensive, and lower MRR. Opus 4.7 ($28.13, MRR 0.730, 2.5h generation) is dominated by Opus 4.5 ($21.51, MRR 0.800, 0.8h generation) (**Supplementary Table 9**): the same vendor, more expensive, slower, and lower MRR on every metric, with the Top-3/Top-5 gap reaching statistical significance.

***Generalizability limitations.*** Several caveats constrain the generalizability of these findings. First, the benchmark scope was limited; $n = 165$ paired queries spread across ten CPTAC studies provide limited statistical resolution. Second, the benchmark was performed over a single domain. The CPTAC studies are exclusively cancer proteomics, and the alias-generation prompt was the cancer_genomics prompt tuned for that domain. Whether the same Tier-A equivalence holds for non-oncology biomedical metadata (e.g., microbiome, EHR, ecology) remains unverified. Cross-domain replication is essential before generalizing the "pick on cost" recommendation outside oncology genomics. Lastly, our statistical tests assumed per-query independence. McNemar's test, the Wilcoxon signed-rank test, and the paired bootstrap all assume independent paired observations. The 66 multi-GT queries ($G > 1$) violate this mildly — they contribute correlated within-query observations to Recall@GT. We accept this as a small bias against the conservative test direction and do not attempt a hierarchical correction.

## Source-side paraphrase reveals a robustness gap in *SchemaMapper*, not an LLM strength

The source-side perturbation (E2) showed the opposite asymmetry to the target-side rename (E3) and identified the one aspect where LLM is more robust than the *SchemaMapper* architecture. Under synonym-swap of source column names, LLM-only lost 7.9–11.5 pp Top-1, whereas every SM+alias configuration lost substantially more (22.4–29.7 pp), and SM-no-alias fell from 53.9 → 33.3% (−20.6 pp, **Supplementary Table 12**): LLMs paraphrase-match degrade more gracefully, while *SchemaMapper*'s embedding retrieval is more sensitive to the lexical form of the source column. Abbreviation-flip followed the same pattern but with smaller magnitudes (LLM-only losses 0.4–6.5 pp; *SchemaMapper* losses 9.1–16.4 pp), consistent with medical abbreviations being sufficiently standardized that both surface forms are equally familiar to both model families and both embedding-based retrievers. Critically, for the SM+alias configurations under E2, the original alias dictionaries were retained unchanged, so these losses reflect the matching stage's sensitivity to source paraphrase rather than degraded alias coverage. This is a limitation of the current *SchemaMapper* retrieval rather than evidence of LLM task competence: unlike the E3 target-rename result, the E2 gap does not invert, and it points to source-side normalization or paraphrase-aware query expansion as a concrete direction for improving *SchemaMapper* robustness.

bioRxiv preprint doi: https://doi.org/10.64898/2026.06.13.732088; this version posted June 17, 2026. The copyright holder for this preprint (which was not certified by peer review) is the author/funder, who has granted bioRxiv a license to display the preprint in perpetuity. It is made available under a CC-BY 4.0 International license.

1. Liu, Y., Pena, E., Santos, A., Wu, E. & Freire, J. Magneto: Combining small and large language models for schema matching. *arXiv [cs.DB]* (2025) doi:10.48550/arXiv.2412.08194.

bioRxiv preprint doi: https://doi.org/10.64898/2026.06.13.732088; this version posted June 17, 2026. The copyright holder for this preprint (which was not certified by peer review) is the author/funder, who has granted bioRxiv a license to display the preprint in perpetuity. It is made available under a CC-BY 4.0 International license.

# Supplementary Figures

## **Supplementary Figure 1.** Choice of LLM model for *SchemaMapper* alias generation

Each alias dictionary was generated by a single LLM and supplied to Stage 1 of *SchemaMapper*, and performance was evaluated on the GDC schema-mapping dataset as in Figure 2. Panels A–C show paired-bootstrap comparisons of each dictionary against the Opus 4.5 dictionary (Δ = method − Opus 4.5), with 95% CIs from B = 10,000 query-matched resamples (n = 165). *Methods whose CI crosses zero (blue) are statistically equivalent to Opus 4.5*; methods whose CI lies entirely below zero (red) are significantly worse. **(A)** ΔTop-1 accuracy. Haiku 4.5, Gemini 2.5 Pro, and Opus 4.7 cross zero, though the Opus 4.7 interval only marginally includes zero. **(B)** ΔTop-5 accuracy. Only Haiku 4.5 was equivalent to Opus 4.5, while all other dictionaries were well below zero. **(C)** ΔMRR. Haiku 4.5 and Gemini 2.5 Pro cross zero. Across panels, the equivalent-to-Opus-4.5 set narrows as the metric becomes more rank-sensitive (Top-1 → MRR → Top-5), with Haiku 4.5 the only dictionary indistinguishable from Opus 4.5 on every metric. **(D)** MRR (95% paired-bootstrap CI) against one-time alias-dictionary generation cost (USD, log scale). The shaded band is the Opus 4.5 95% CI, and the dashed line is the no-alias baseline. Point shape denotes hosting (circle = API, triangle = local) and color denotes vendor (orange = Anthropic, blue = Google). The dotted line traces the cost–performance Pareto frontier (Gemini 2.5 Flash → Haiku 4.5 → Gemini 2.5 Pro → Opus 4.5); Sonnet 4.5 and Opus 4.7 sit off-frontier, each dominated by a cheaper same-vendor alternative.

**A.**

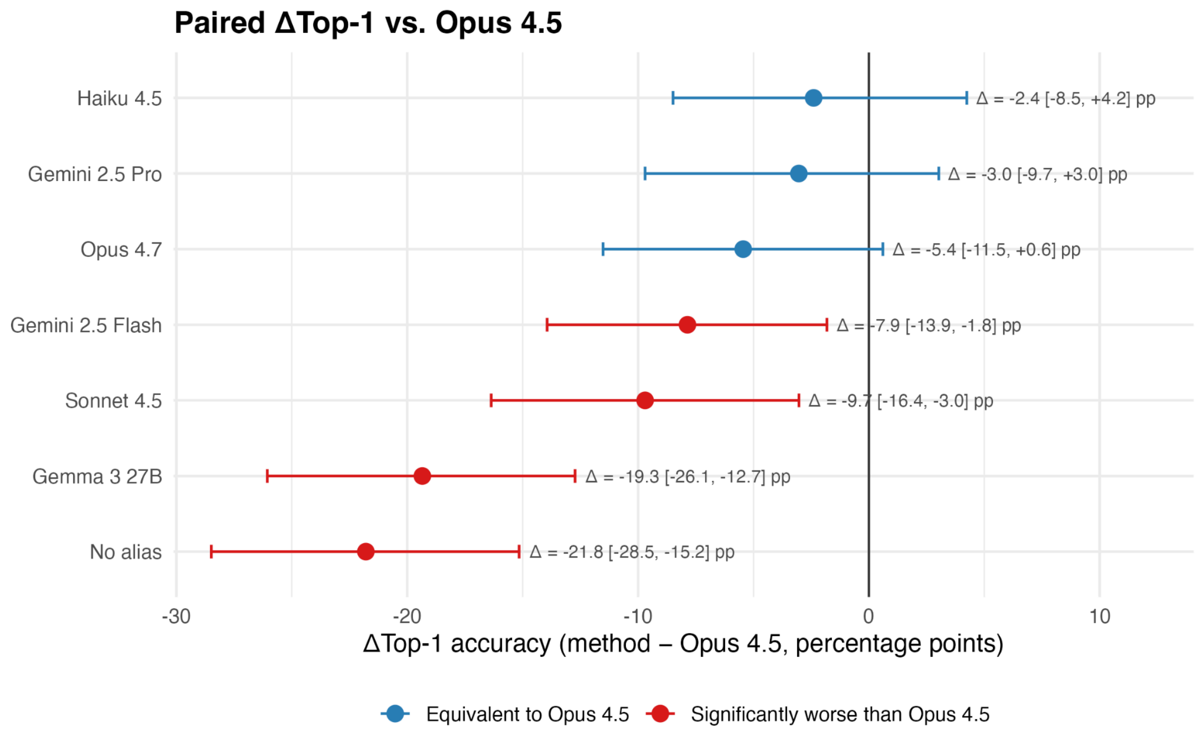

bioRxiv preprint doi: https://doi.org/10.64898/2026.06.13.732088; this version posted June 17, 2026. The copyright holder for this preprint (which was not certified by peer review) is the author/funder, who has granted bioRxiv a license to display the preprint in perpetuity. It is made available under a CC-BY 4.0 International license.

**B.**

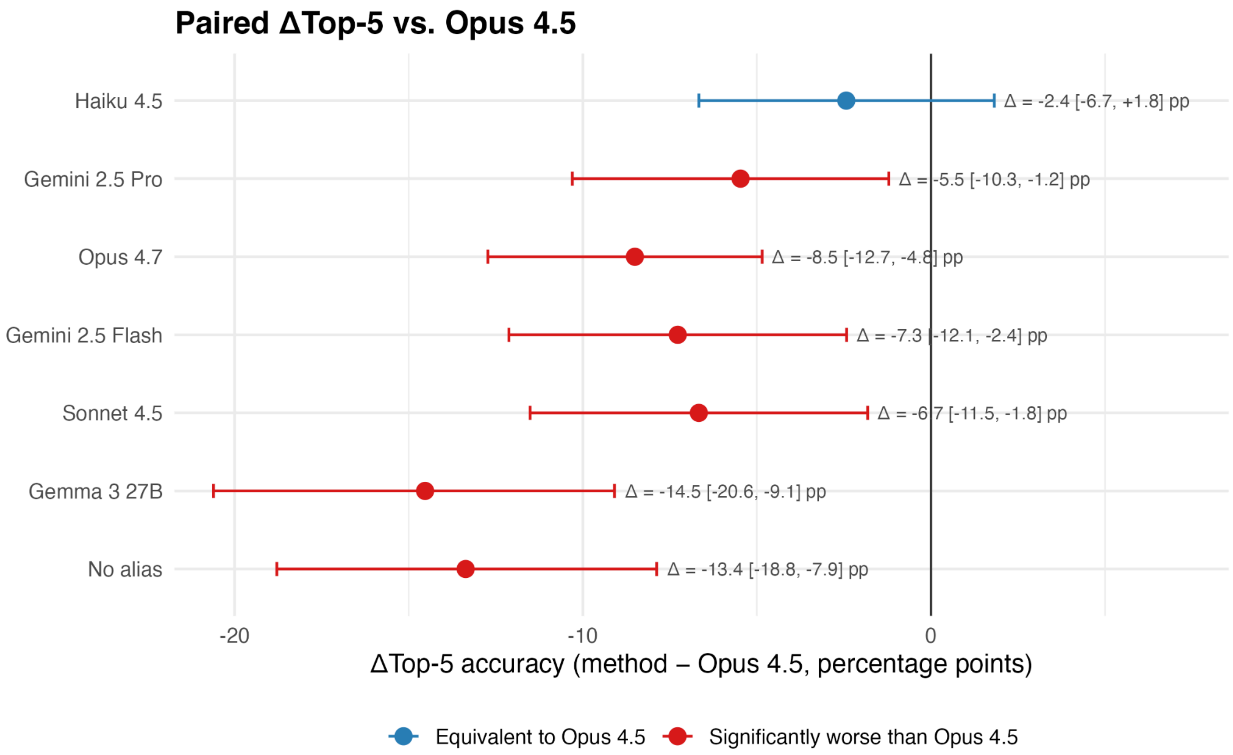


**C.**

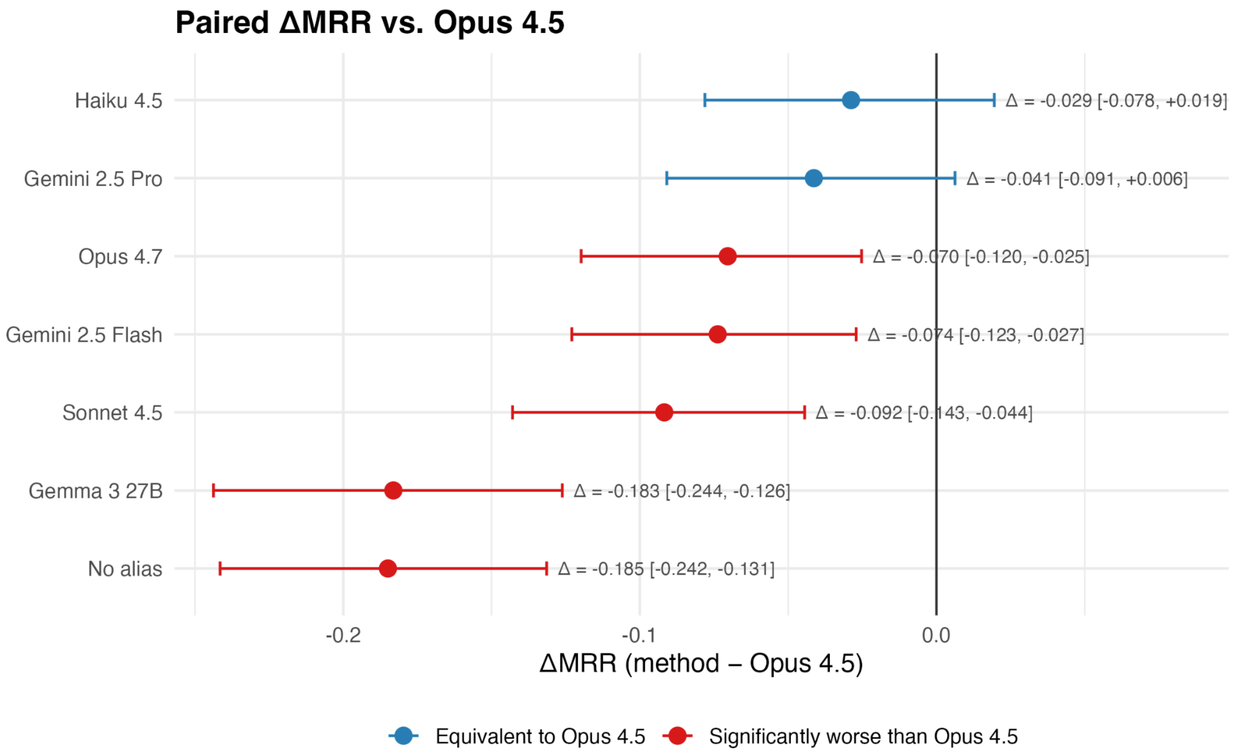

bioRxiv preprint doi: https://doi.org/10.64898/2026.06.13.732088; this version posted June 17, 2026. The copyright holder for this preprint (which was not certified by peer review) is the author/funder, who has granted bioRxiv a license to display the preprint in perpetuity. It is made available under a CC-BY 4.0 International license.

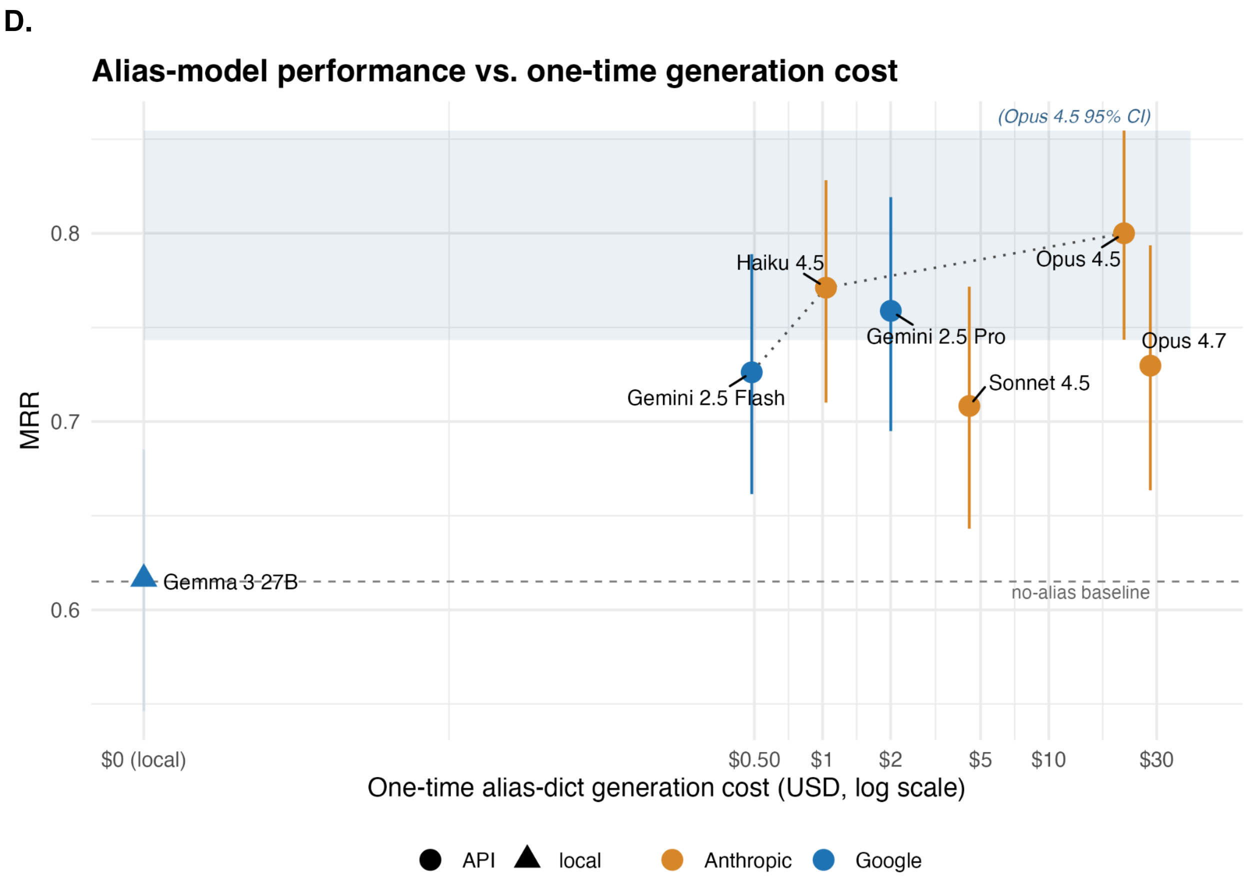

# Supplementary Tables

**Supplementary Table 1.** *SchemaMapper* configuration parameters

Per-stage thresholds, methods, and acceptance rules. All embedding-based methods use the `all-MiniLM-L6-v2` sentence encoder by default; Stage 2 ontology matching additionally uses the NCI Thesaurus API. Cascade behavior across stages: each method accepts when its Top-1 score meets the threshold; otherwise, the best-seen candidate is retained and the cascade advances. Sub-threshold results are still returned if no better match is found.

| Stage | Method | Metric | Threshold | Score formula | Notes |
|---|---|---|---|---|---|
| Pre-processing | normalization | rule-based | — | — | Lowercase + punctuation/whitespace cleanup applied to all column names and dictionary keys. |
| 1 | std_exact / alias_exact | exact match | 1 | 1 | alias_exact runs only when an alias dictionary is loaded. |
| 1 | std_fuzzy / alias_fuzzy | RapidFuzz token_sort_ratio | 92 | ratio / 100 | Per-field max kept across alias matches. |
| 2-1 | value | per-value cosine + log-freq-weighted proportion | 0.85 (per-value); 0.2 (proportion) | log-freq weighted proportion | Skipped for numeric columns and for non-enumerable columns (e.g., IDs). Per-value top-10 candidates filtered before aggregation. Weight = log1p(freq · 10). |
| 2-2 | ontology | log-freq-weighted proportion | 0.2 | proportion (+0.3 if column name maps; capped at 1.0) | NCI Thesaurus API. |
| 3-1 | numeric | cosine similarity | 0.6 | cosine + family_boost | Runs only for numeric columns. Keys normalized; units and tags stripped before matching; family detected via detect_numeric_semantic. |
| 3-2 | semantic | cosine similarity | 0.5 | cosine | Combined std + alias index, max per field. Alias side runs only when the alias dictionary is loaded. |
| Global | top_k | count | 5 | — | Limits the number of candidates returned per column at every stage; thresholds unaffected. |

**Supplementary Table 2**. *OntologyMapper* configuration parameters

Per-stage thresholds, methods, and acceptance rules. Embeddings use SapBERT (`cambridgeltl/SapBERT-from-PubmedBERT-fulltext`) by default. All FAISS indices are `IndexFlatIP` over L2-normalized vectors, making the inner product equivalent to cosine similarity. Cascade behavior: Stage 1 exact matches bypass all later stages; non-exact queries enter Stage 2 unconditionally; Stage 2.5 is invoked only when the Stage 2 Top-1 score falls below the synonym-boost threshold.

| Stage | Method | Metric | Threshold | Score formula | Notes |
|---|---|---|---|---|---|
| 1 | exact | case-insensitive string match | 1 | 1 | .strip().lower() against corpus set. |
| 2 | lm (default) | FAISS inner product | — | FAISS IP | CLS-token embedding via HuggingFace AutoModel; max_length = 128. |
| 2 | st | FAISS inner product (cosine-equivalent) | — | cosine | SentenceTransformer pooling per checkpoint; normalize_embeddings = True. |
| 2.5 | synonym boost | synonym lookup score | 0.9 | max(Stage 2 score, synonym score) per term | Targets Stage 2 rows with top-1 < 0.9. Merges Stage 2 and synonym candidates, max score per term, re-ranks Top-k. Stage label updated to 2.5 only if boosted (new Top-1 > old Top-1, or match_level improves). Synonym tables sourced from NCI EVSREST or OLS per ontology_source. |
| Global | top_k | count | 5 | — | Limits the number of returns per query at every stage. |
| Global | filter_obsolete | prefix filter | False (default) | — | When True, drops obsolete_* labels from both corpus and synonym candidates. |
| Global | corpus registry | lookup | — | — | Auto-resolves (category, ontology_source) to root term; e.g., (disease, ncit) → NCIT:C3262. Other combinations require a user-supplied corpus. |

**Supplementary Table 3.** *SchemaMapper* performance under different alias-dictionary configurations

We compared the performance of five alias variants for schema mapping across 10 CPTAC studies. Variants differ only in the alias dictionary supplied to Stage 1 of the *SchemaMapper* pipeline: seven LLM-generated dictionaries and a no-alias control on the raw column names only. All metrics are per-query means over the 165 ground-truth mappings (case-insensitive matching). Micro-average is used here; by contrast, Table 1 uses macro-averaging to match the Magneto convention. SM = *SchemaMapper.*

| Method | Top-1 (%) | Top-3 (%) | Top-5 (%) | MRR (micro) | Recall@GT |
|---|---|---|---|---|---|
| SM-only (no alias) | 53.94 | 69.7 | 72.73 | 0.615 | 0.535 |
| SM + Haiku 4.5 alias | 73.33 | 81.21 | 83.64 | 0.771 | 0.676 |
| SM + Sonnet 4.5 alias | 66.06 | 76.36 | 79.39 | 0.708 | 0.635 |
| SM + Opus 4.5 alias | 75.76 | 83.64 | 86.06 | 0.8 | 0.699 |
| SM + Opus 4.7 alias | 70.3 | 75.15 | 77.58 | 0.73 | 0.643 |
| SM + Gemini 2.5 Flash alias | 67.88 | 76.97 | 78.79 | 0.726 | 0.626 |
| SM + Gemini 2.5 Pro alias | 72.73 | 80 | 80.61 | 0.759 | 0.66 |
| SM + Gemma 3 27B alias | 56.36 | 63.64 | 71.52 | 0.616 | 0.505 |

**Supplementary Table 4.** *SchemaMapper* performance by pipeline stage and method, with or without LLM-generated aliases

Each row reports the number of queries (*n*) resolved at a given stage/method combination across all 10 CPTAC studies (165 queries total), along with the corresponding accuracy at rank 1, 3, and 5 (Top-k) and Mean Reciprocal Rank (MRR). Invalid queries (4 for Stage 1 and 13 for Stage 3) contribute zeros to all accuracy metrics. The "Haiku-4.5 alias" configuration adds a Haiku 4.5–generated alias dictionary on top of the target dictionary; "No alias" uses the target dictionary only. Adding aliases reroutes 30 queries from Stage 3 to Stage 1 (27 alias_exact + 3 alias_fuzzy) and improves the residual Stage 3 semantic retrieval (Top-1 38.6% → 62.1%; MRR 0.495 → 0.692), indicating that LLM aliases both deflect easy cases away from the semantic stage and leave a more tractable residual for embedding-based matching. We used the Top-5 retrievals and the micro-average for this evaluation.

| Configuration | Stage | Method | n | Top-1 (%) | Top-3 (%) | Top-5 (%) | MRR (micro) |
|---|---|---|---|---|---|---|---|
| Haiku-4.5 alias | Stage 1 | std_exact | 46 | 95.7 | 95.7 | 95.7 | 0.957 |
| | Stage 1 | std_fuzzy | 1 | 100 | 100 | 100 | 1 |
| | Stage 1 | alias_exact | 27 | 81.5 | 81.5 | 81.5 | 0.815 |
| | Stage 1 | alias_fuzzy | 3 | 0 | 0 | 0 | 0 |
| | Stage 2 | value/ontology | 1 | 0 | 0 | 0 | 0 |
| | Stage 3 | semantic | 87 | 62.1 | 77 | 81.6 | 0.692 |
| No alias | Stage 1 | std_exact | 46 | 95.7 | 95.7 | 95.7 | 0.957 |
| | Stage 1 | std_fuzzy | 1 | 100 | 100 | 100 | 1 |
| | Stage 2 | value/ontology | 4 | 0 | 0 | 0 | 0 |
| | Stage 3 | semantic | 114 | 38.6 | 61.4 | 65.8 | 0.495 |

**Supplementary Table 5.** Per-stage accuracy of *OntologyMapper* on the four EFO benchmarks

Each query is attributed to the pipeline stage that produced its Top-1 prediction. Per-stage *n* are output-row counts (see Denominator conventions, **Supplementary Methods**); these differ slightly from the unique-query denominators used in Table 2, so per-stage and overall accuracies rest on marginally different bases. Stage 1 is exact-match by construction, so its Top-K is 100%. We used the Top-5 retrievals and the micro-average for this evaluation.

| Benchmark | Stage | n | Top-1 (%) | Top-3 (%) | Top-5 (%) | MRR (micro) |
|---|---|---|---|---|---|---|
| UKBB-EFO | 1 | 288 | 100 | 100 | 100 | 1 |
| | 2 | 284 | 70.42 | 82.04 | 84.51 | 0.763 |
| | 2.5 | 318 | 65.41 | 80.82 | 85.22 | 0.733 |
| Biomappings-EFO | 1 | 576 | 100 | 100 | 100 | 1 |
| | 2 | 127 | 85.04 | 94.49 | 97.64 | 0.906 |
| | 2.5 | 92 | 81.52 | 92.39 | 92.39 | 0.861 |
| OLS-EFO (disease) | 1 | 4,014 | 100 | 100 | 100 | 1 |
| | 2 | 1,206 | 88.64 | 95.52 | 96.77 | 0.921 |
| | 2.5 | 584 | 75.68 | 83.05 | 85.79 | 0.796 |
| OLS-EFO (full) | 1 | 4,825 | 100 | 100 | 100 | 1 |
| | 2 | 1,552 | 76.1 | 82.02 | 83.18 | 0.791 |
| | 2.5 | 1,068 | 59.18 | 67.23 | 69.57 | 0.634 |

**Supplementary Table 6.** Confidence calibration of *OntologyMapper* Top-1 similarity scores across four EFO benchmarks

For each benchmark, queries are partitioned into correctly and incorrectly mapped at Top-1 (n correct, n incorrect). Mean conf. (correct) and Mean conf. (incorrect) are the average Top-1 cosine similarities in each group; Δ is their difference (correct − incorrect). Cohen's d quantifies the standardized separation between the two confidence distributions (pooled SD), and AUC is the area under the ROC curve for using the Top-1 score as a classifier of mapping correctness (0.5 = chance, 1.0 = perfect separation). Larger Δ, |d|, and AUC indicate that the Top-1 score is more informative as a confidence signal (i.e., better-calibrated). All four benchmarks use the baseline configuration (Stages 1/2/2.5 with SapBERT embeddings). Wilcoxon rank-sum p-values were $< 2 \times 10^{-16}$ for all rows (Holm-adjusted). We used the Top-5 retrievals and the micro-average for this evaluation.

| Benchmark | n correct | n incorrect | Mean conf. (correct) | Mean conf. (incorrect) | Δ | Cohen's d | AUC |
|---|---|---|---|---|---|---|---|
| UKBB-EFO | 694 | 196 | 0.943 | 0.86 | 0.0829 | 0.954 | 0.766 |
| OLS-EFO (full) | 6,638 | 807 | 0.988 | 0.851 | 0.1368 | 2.579 | 0.895 |
| OLS-EFO (disease) | 5,525 | 279 | 0.99 | 0.83 | 0.1602 | 3.89 | 0.935 |
| Biomappings-EFO | 759 | 36 | 0.986 | 0.871 | 0.1148 | 2.804 | 0.917 |

**Supplementary Table 7.** *OntologyMapper* runtime comparison between cold (first-time) and warm (cached) runs

We measured *OntologyMapper's* runtime on the 33,230-term EFO corpus. Cold and warm runs are defined in the Methods section. ***Init***: concept table construction time. ***Model Init***: time to load SapBERT model weights from the local cache directory into memory. ***Inference***: Stage 2 SapBERT forward pass, comprising query embedding and FAISS nearest-neighbor search against the corpus. ***Pipeline***: cumulative wall-clock time across all mapping stages (normalization, Stages 1, 2, and 2.5), excluding initialization. ***Total***: Init + Pipeline, representing the full end-to-end runtime from invocation to completion. All times are reported in seconds.

| Run | Benchmark | n | Init (s) | Model Init (s) | Inference (s) | Pipeline (s) | Total (s) |
|---|---|---|---|---|---|---|---|
| Cold (first) | UKBB-EFO | 888 | **1168.3** | NA | NA | 11.7 | 1180 |
| Cold (first) | Biomappings-EFO | 795 | **1342.9** | NA | NA | 4.1 | 1347 |
| Cold (first) | OLS-EFO (disease) | 5824 | **1290.4** | NA | NA | 28.6 | 1319 |
| Cold (first) | OLS-EFO (full) | 7504 | **1279.1** | NA | NA | 42.9 | 1322 |
| Warm (cached) | UKBB-EFO | 888 | **0.36** | 0 | 9.71 | 11.75 | 12.11 |
| Warm (cached) | Biomappings-EFO | 795 | **0.33** | 0 | 3.37 | 4.12 | 4.45 |
| Warm (cached) | OLS-EFO (disease) | 5824 | **0.31** | 0.01 | 25.31 | 28.57 | 28.89 |
| Warm (cached) | OLS-EFO (full) | 7504 | **0.3** | 0 | 37.18 | 42.9 | 43.2 |

**Supplementary Table 8.** Wall-clock runtime of *OntologyMapper* and *text2term* across four EFO benchmarks

Per-benchmark execution time (mm:ss) for *OntologyMapper* across two operating modes, compared with *text2term* (TF-IDF baseline). *Cold* denotes the first run against a fresh corpus, including one-time construction of the embedding index over a 12-ontology EFO corpus (33,230 terms for *OntologyMapper* and 33,659 terms for *text2term*) parsed from a local OWL file and FAISS index serialization to disk. *Warm* denotes subsequent runs that reuse the cached FAISS index and SQLite metadata store, reflecting the steady-state cost users incur after initial setup. *text2term* (TF-IDF) timings include the local OWL parse; the original *text2term* evaluation pulled the OWL over HTTP, adding ~5s per benchmark. *n* queries indicate the number of input strings mapped per benchmark. Totals are summed across all four benchmarks (15,011 queries). All measurements were collected on a single workstation: Apple M4 Pro, 48 GB unified memory, internal SSD, macOS Sequoia 15.6.1 (aarch64), with no concurrent workloads. Cold-mode cost is incurred once per (model, corpus-content) pair and is excluded from the typical end-user runtime once the index is cached.

| Benchmark | n queries | Cold | Warm | text2term |
|---|---|---|---|---|
| UKBB-EFO | 888 | 19m 40s | 12.1s | 14.4s |
| Biomappings-EFO | 795 | 22m 27s | 4.5s | 13.8s |
| OLS-EFO (disease) | 5824 | 21m 59s | 28.9s | 14.8s |
| OLS-EFO (full) | 7504 | 22m 02s | 43.2s | 15.3s |
| **Total** | **15,011** | **1h 26m 08s** | **1m 28.7s** | **58.3s** |

**Supplementary Table 9.** Summary of LLM-generated alias dictionaries for the 736-field GDC schema

Nine large language models were evaluated for their ability to generate alias dictionaries for the 736 GDC-standard fields used in the *SchemaMapper* benchmark. Four Anthropic models (Claude Haiku 4.5, Sonnet 4.5, Opus 4.5, Opus 4.7), two Google API models (Gemini 2.5 Flash, Gemini 2..5 Pro), and three locally hosted open-weight models (Gemma3:27b, Gemma4:26b, Qwen3:32b) were run with an identical five-pass prompt template; no prompt-level adjustments were made per model. Rows is the total number of (field, alias) entries in the resulting dictionary after format validation and, for Gemma3:27b, post-hoc removal of hallucinated field names. Aliases per field are computed as Rows / 736, except for Claude Haiku 4.5, where the reported value reflects the per-field mean after de-duplication. Wall time is end-to-end generation time on the production hardware (Anthropic API for Claude models; local GPU for open-weight models). API cost is the total amount billed by the provider; locally run models incur no API charge. Failed runs (Gemma4:26b: malformed output that could not be parsed; Qwen3:32b: structurally valid output in which the source-alias column contained the literal placeholder labels A–F from the few-shot example, a "template capture" failure mode) produced no usable dictionary and are reported with em-dashes. All completed dictionaries achieved 100% field coverage.

| Model | Provider / Mode | Status | # of Alias | Aliases per field | Top-1 (%) | Wall time | API cost (USD) | Notes |
|---|---|---|---|---|---|---|---|---|
| Claude Haiku 4.5 | Anthropic API | Completed | 9,219 | 12.4 | 73.33 | 16.5 min | ~$1.04 | |
| Claude Sonnet 4.5 | Anthropic API | Completed | 14,048 | 19.1 | 66.06 | 49 min | $4.45 | |
| Claude Opus 4.5 | Anthropic API | Completed | 14,200 | 19.3 | 75.76 | 46 min | ~$21.51 | |
| Claude Opus 4.7 | Anthropic API | Completed | 12,515 | 17 | 70.3 | 2 h 31 min | $28.13 | |
| Gemini 2.5 Flash | Google API | Completed | 9,440 | 13 | 67.88 | 13.4 min | ~$0.49 | 151 rows from 7 hallucinated fields filtered (1.3% of raw) |
| Gemini 2.5 Pro | Google API | Completed | 10,756 | 15 | 72.73 | 41 min | ~$2.00 | 2 rows from 2 hallucinated fields filtered (0.02% of raw) |
| Gemma3:27b | Open-weight (local) | Completed | 7,757 | 10.5 | 56.36 | 4 h 41 min | $0.00 | 185 rows from 105 hallucinated fields filtered (1.9% of raw) |
| Gemma4:26b | Open-weight (local) | Failed | — | — | — | — | — | Failed: format compliance issue |
| Qwen3:32b | Open-weight (local) | Failed | — | — | — | — | — | Failed: template capture (literal A–F from few-shot example) |

**Supplementary Table 10.** LLM-only baseline vs *SchemaMapper* on the GDC schema-mapping benchmark

We compared the schema-mapping performance of different configuration families on 165 source-to-target column mappings drawn from 10 CPTAC proteomics studies against the 736-column GDC clinical data model. Only the source and target column names were used. Three configuration families are: (i) *SchemaMapper* without alias enrichment (Stages 1–3); (ii) *SchemaMapper* with LLM-generated alias dictionaries (Stages 1–3 plus a Stage-1 alias dictionary of 9,000–14,000 entries, one dictionary per LLM family); and (iii) LLM-only, in which the source column is sent to a frontier LLM together with the full 736-column target list in a single zero-shot call and the Top-5 predictions are extracted from the JSON response. 95% confidence intervals are 1,000-replicate bootstrap intervals over the 165 queries. '*Δ vs SM-no-alias*' gives the absolute Top-1 difference against the no-alias baseline. '*Δ vs matched-model SM+alias*' compares each LLM-only row to the *SchemaMapper* hybrid that uses the corresponding alias dictionary. Claude Opus 4.7 has no LLM-only row (see Methods). We used the Top-5 retrievals and the *micro-average* for this evaluation. SM = *SchemaMapper*.

| Method | Top-1 [95% CI] | MRR (micro) | Δ vs SM-no-alias | Δ vs matched-model SM+alias |
|---|---|---|---|---|
| SM (no alias) | 53.94% | 0.615 | — | — |
| SM + Haiku 4.5 alias | 73.33% | 0.771 | +19.4 pp | — |
| SM + Sonnet 4.5 alias | 66.06% | 0.708 | +12.1 pp | — |
| SM + Opus 4.5 alias | 75.76% | 0.8 | +21.8 pp | — |
| SM + Opus 4.7 alias | 70.30% | 0.73 | +16.4 pp | — |
| SM + Gemini 2.5 Flash alias | 67.88% | 0.726 | +13.9 pp | — |
| SM + Gemini 2.5 Pro alias | 72.73% | 0.759 | +18.8 pp | — |
| Claude Haiku 4.5 | 83.6% [78.2, 89.1] | 0.877 | +29.7 pp | +10.3 pp |
| Claude Sonnet 4.5 | 89.7% [84.8, 94.5] | 0.932 | +35.8 pp | +23.6 pp |
| Claude Opus 4.5 | 86.7% [81.2, 91.5] | 0.916 | +32.8 pp | +10.9 pp |
| Gemini 2.5 Flash | 83.0% [77.0, 88.5] | 0.876 | +29.1 pp | +15.1 pp |
| Gemini 2.5 Pro | 89.1% [84.2, 93.3] | 0.925 | +35.2 pp | +16.4 pp |

**Supplementary Table 11.** Direct memorization probes (E4) across five frontier LLMs

Three complementary probes assess whether each model has memorized the GDC clinical schema. P1 (demographic-node recall): when prompted to enumerate the columns of the GDC demographic node, the percentage of generated lines whose first emitted token matches a valid GDC field name; parentheses report (valid hits / total lines emitted). Haiku 4.5 produced only five generic placeholders rather than schema-grounded column names. P2 (reverse concept → GDC): exact-match rate when the model is given 20 clinical concept descriptions in isolation (no schema context) and asked to return the canonical GDC identifier; parentheses report (correct / 20). P3 (random 3-slot recovery): across ten 12-name slices of the GDC schema, three names per slice are masked at random positions, and the model is asked to recover them; the reported value is blank-level pooled exact-match recall — total correctly recovered names divided by 30 total blanks (10 slices × 3 masks per slice), order-independent within each slice. Higher values across all three probes indicate stronger memorization of GDC schema content; P3 is the most stringent probe, requiring positional recovery rather than recognition.

| Model | P1 (demographic-node recall) | P2 (reverse concept → GDC) | P3 (random 3-slot) |
|---|---|---|---|
| Claude Haiku 4.5 | 0% (5 unspecific lines) | 65% (13/20) | 0% |
| Claude Sonnet 4.5 | 88% (15/17) | 100% (20/20) | 0% |
| Claude Opus 4.5 | 95% (18/19) | 100% (20/20) | 0% |
| Gemini 2.5 Flash | 83% (5/6) | 65% (13/20) | 0% |
| Gemini 2.5 Pro | 92% (12/13) | 80% (16/20) | 7% |

**Supplementary Table 12.** Matched-control perturbation battery on the GDC benchmark

All configurations are evaluated on the 165-query benchmark (the same as that used in the *SchemaMapper* benchmark) under three perturbations: the original benchmark (E1), source-side variants (E2) reported separately (i.e., synonym-swap and abbreviation-flip), and target-side rename (E3). For '*SchemaMapper* (SM)+alias' under E3, the alias dictionary is translated through the rename map, so aliases continue to point at valid targets. LLM-only baseline values are the names-only configuration. We used the Top-5 retrievals and the micro-average for this evaluation.

| Configuration | E1 baseline | E2 synonym-swap (Δ) | E2 abbrev-flip (Δ) | E3 target-rename (Δ) |
|---|---|---|---|---|
| SM-no-alias | 53.90% | 33.3% (−20.6) | 35.8% (−18.2) | 40.6% (−13.3) |
| SM + Haiku 4.5 alias | 73.30% | 43.6% (−29.7) | 60.0% (−13.3) | 71.5% (−1.8) |
| SM + Sonnet 4.5 alias | 66.10% | 36.4% (−29.7) | 55.2% (−10.9) | 68.5% (+2.4) |
| SM + Opus 4.5 alias | 75.80% | 50.9% (−24.9) | 59.4% (−16.4) | 78.8% (+3.0) |
| SM + Opus 4.7 alias | 70.30% | 45.5% (−24.8) | 61.2% (−9.1) | 68.5% (−1.8) |
| SM + Gemini 2.5 Flash alias | 67.90% | 42.4% (−25.5) | 52.7% (−15.2) | 70.3% (+2.4) |
| SM + Gemini 2.5 Pro alias | 72.70% | 50.3% (−22.4) | 56.4% (−16.4) | 75.2% (+2.4) |
| LLM-only Haiku 4.5 | 83.60% | 75.2% (−8.5) | 83.2% (−0.4) | 64.2% (−19.4) |
| LLM-only Sonnet 4.5 | 89.70% | 78.2% (−11.5) | 83.2% (−6.5) | 66.1% (−23.6) |
| LLM-only Opus 4.5 | 86.70% | 77.6% (−9.1) | 85.1% (−1.6) | 74.5% (−12.1) |
| LLM-only Gemini 2.5 Flash | 83.00% | 75.2% (−7.9) | 80.7% (−2.3) | 64.8% (−18.2) |
| LLM-only Gemini 2.5 Pro | 89.10% | 80.0% (−9.1) | 85.7% (−3.4) | 71.5% (−17.6) |

**Supplementary Table 13.** LLM-only baselines fall short of *OntologyMapper* on the UKBB–EFO benchmark

The ontology-mapping performance of 888 UKBB-EFO benchmark queries was evaluated across three methods: *OntologyMapper*, LLM-only *zero-shot*, and LLM-only *open_book_full*. The LLM-only approach prompts Claude Haiku 4.5 (`temperature = 0`) to return the Top-5 EFO labels. For *zero-shot*, no candidate list was provided, and for *open_book_full*, the full EFO label corpus is provided in context. All three methods were evaluated against the same 17,638-label EFO corpus; the 33,230-label EFO corpus used in the *text2term* benchmarking exceeds Haiku 4.5's input limit and was therefore not used here. 'Halluc. (Top-1)' is the fraction of queries whose Top-1 prediction is absent from the corpus. We used the Top-5 retrievals and the micro-average for this evaluation.

| Method | Top-1 (%) | Top-3 (%) | Top-5 (%) | MRR (micro) | Halluc. (Top-1, %) |
|---|---|---|---|---|---|
| *OntologyMapper* | 82.98 | 93.95 | 96.75 | 0.887 | — |
| LLM-only, zero_shot | 54.88 | 61.84 | 63.08 | 0.584 | 34.46 |
| LLM-only, open_book_full | 63.75 | 71.04 | 73.06 | 0.676 | 5.95 |

**Supplementary Table 14.** Evaluation metrics and applicable aggregation schemes

Filled circles (●) indicate aggregation schemes reported in this study. Em-dashes (—) indicate schemes that are not applicable because pooled aggregation coincides with micro-averaging for per-query metrics and so is not reported separately. "n/a" indicates schemes that are undefined or degenerate for the metric: micro-averaging is not defined for global Recall@GT, which produces a single study-level value by construction; both micro and macro are degenerate for confidence calibration metrics on small studies. Cross-method comparisons in Table 1 use macro averaging throughout to match the convention used by Liu et al. in the source Magneto evaluation.

| **Metric** | **Micro** pool queries across studies | **Macro** average study-level values | **Pooled** all predictions |
|---|---|---|---|
| Top-k accuracy (k = 1-5) | ● | ● | — |
| Mean Reciprocal Rank (MRR) | ● | ● | — |
| Recall@GT, per-query | ● | ● | — |
| Recall@GT, global | n/a | ● | — |
| Confidence calibration | n/a | n/a | ● |

**Supplementary Table 15.** Practical guide to LLM model selection for *SchemaMapper* alias generation

Decision-support summary for the seven alias dictionaries evaluated on the GDC schema-matching benchmark, ranked by MRR within performance tiers. For each model, the table reports vendor, hosting mode (API vs. local), MRR with 95% paired-bootstrap CI, Top-1 accuracy, one-time dictionary-generation cost (USD; $0 for locally hosted models), wall-clock generation time, and a recommended use case. Tier-A comprises the six dictionaries statistically equivalent to one another on Top-1 accuracy (all-pairs McNemar, Holm-adjusted over 28 comparisons); within this tier, point estimates favor Opus 4.5, but Haiku 4.5 is statistically indistinguishable from Opus 4.5 on every metric at roughly 20× lower cost and ~2.8x faster generation time, making it the recommended default. Gemini 2.5 Flash is the lowest-cost option, and Gemini 2.5 Pro is the vendor-diversified choice; Sonnet 4.5 and Opus 4.7 are Pareto-dominated and not recommended for new runs. Tier B (Gemma 3 27B) is a local option with no measurable benefit over no-alias on this benchmark. Tier C is the no-alias baseline. Costs reflect public list prices as of late 2025/early 2026 and exclude batch-API or prompt-caching discounts.

| Method | Vendor | Hosting | MRR | Top-1 (%) | One-time cost | Gen. time | When to choose |
|---|---|---|---|---|---|---|---|
| ***Tier A (statistically equivalent on MRR / Top-1)*** | | | | | | | |
| Opus 4.5 | Anthropic | API | 0.800 [0.743, 0.855] | 75.76 | $21.51 | 0.8 h | Max accuracy; cost is no constraint. |
| Haiku 4.5 | Anthropic | API | 0.771 [0.710, 0.828] | 73.33 | $1.04 | 0.3 h | **Default recommendation — statistically equivalent to Opus 4.5 at ~20× lower cost and ~2.8x faster.** |
| Gemini 2.5 Pro | Google | API | 0.759 [0.695, 0.819] | 72.73 | $2.00 | 0.7 h | Vendor-diverse alternative to Anthropic at low cost. |
| Opus 4.7 | Anthropic | API | 0.730 [0.664, 0.794] | 70.30 | $28.13 | 2.5 h | Dominated by Opus 4.5 (same vendor, more expensive, worse MRR). Avoid for new runs. |
| Gemini 2.5 Flash | Google | API | 0.726 [0.662, 0.789] | 67.88 | $0.49 | 0.2 h | Cheapest API option; equivalent to Tier A on MRR. |
| Sonnet 4.5 | Anthropic | API | 0.708 [0.643, 0.772] | 66.06 | $4.45 | 0.8 h | Dominated by Haiku 4.5 (same vendor, more expensive, similar MRR). Avoid for new runs. |
| ***Tier B (below Tier A)*** | | | | | | | |
| Gemma 3 27B | Google | local | 0.616 [0.546, 0.685] | 56.36 | $0 (local) | 4.7 h | Air-gapped/on-prem only. No measurable benefit over no-alias on this benchmark. |
| ***Tier C (baseline)*** | | | | | | | |
| No alias | NA | NA | 0.615 [0.548, 0.682] | 53.94 | $0 (none) | — | Baseline only. Tier-A alias can add ~12–19 pp Top-1 at <$5. |
